# Catechol-functionalized carbon nanofibers enabling ultrafine CoFe_2_O_4_ nanorods for high-performance bifunctional oxygen electrocatalysis and Zn–air batteries

**DOI:** 10.1039/d6ra09690e

**Published:** 2026-11-04

**Authors:** Rabiya Javed Awan, Usman Hayder, Senem Çitoğlu, Hatice Duran, Salman Noshear Arshad

**Affiliations:** a Department of Chemistry and Chemical Engineering, Syed Babar Ali School of Science and Engineering, Lahore University of Management Sciences Lahore 54792 Pakistan 20130026@lums.edu.pk salman.arshad@kfupm.edu.sa; b Helmholtz Institute Ulm (HIU), Electrochemical Energy Storage Helmholtzstrasse 11 89081 Ulm Germany; c Department of Materials Science & Nanotechnology Engineering, TOBB University of Economics and Technology Söğütözü Cad. 43 Ankara 06560 Türkiye; d UNAM Institute of Materials Science and Nanotechnology, Bilkent University 06800 Ankara Türkiye; e Department of Materials Science and Engineering, King Fahd University of Petroleum & Minerals Dhahran 31261 Saudi Arabia; f Interdisciplinary Research Center for Hydrogen Technologies and Carbon Management, King Fahd University of Petroleum & Minerals Dhahran 31261 Saudi Arabia

## Abstract

The interfacial mismatch between metal oxide and carbon support remains a fundamental bottleneck in bifunctional oxygen electrocatalysts, often leading to poor charge transfer, structure instability and underutilized active sites. In this work, we report the controlled anchoring of ultrafine cobalt ferrite (CoFe_2_O_4_) nanorods on catechol functionalized porous carbon nanofibers (PCNF), denoted as CoFe_2_O_4_@DP@PCNF. Surface functionalization with catechol groups *via* dopamine linkages effectively regulates the nucleation of CoFe_2_O_4_, resulting in a substantial reduction of the nanorod size (from ∼103 to ∼29 nm) and a homogeneous dispersion across the conductive carbon network. Benefiting from the ultra-fine size and strong spinel oxide–carbon interfacial coupling, the optimized hybrid catalyst delivers a half-wave potential of 0.77 V *vs.* RHE for ORR, comparable to commercial Pt/C (0.79 V), and achieves a current density of 10 mA cm^−2^ at an overpotential of 301 mV for OER, outperforming RuO_2_ (410 mV). When applied as the air electrode in a rechargeable Zn–air battery, CoFe_2_O_4_@DP@PCNF exhibits an open-circuit voltage of 1.372 V and excellent cycling stability for 110 h. This work demonstrates an effective surface functionalized strategy for regulating particle size and provides an advanced pathway for designing high-performance oxygen electrocatalysts.

## Abstract 2

Catechol functionalization enables uniform anchoring of ultrafine CoFe_2_O_4_ nanorods on porous carbon nanofibers, strengthening oxide-carbon interfacial interaction and promoting bifunctional ORR/OER activity for durable rechargeable Zn–air batteries.

## Graphical abstract

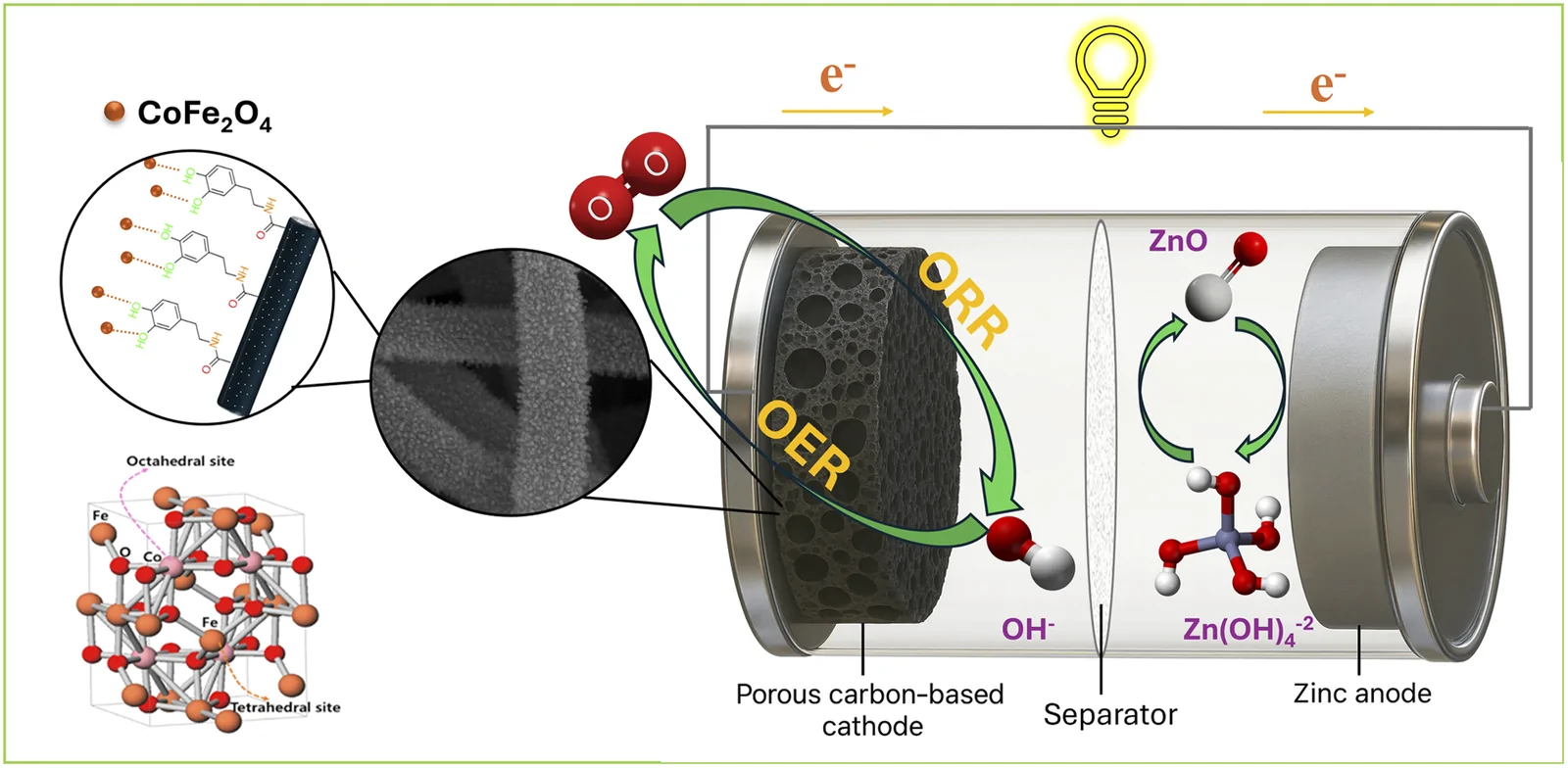

## Introduction

The transition to sustainable energy systems has accelerated rapidly, driven by the necessity to lessen dependence on fossil fuels and mitigate adverse effects of climate change.^1–3^ With renewable energy deployment expanding and global energy storage capacity expected to exceed 2 TWh by 2030, the development of efficient, reliable, and cost-effective energy storage technologies has become imperative.^4,5^ Although lithium-ion batteries dominate the market, challenges such as resource scarcity, supply chain vulnerabilities, safety concerns related to thermal runaway, and escalating material costs have prompted the search for alternative electrochemical energy storage systems.^6–8^ Among them, zinc–air batteries (ZABs) are promising due to their high theoretical energy density (1086 Wh kg^−1^), practical energy density (500–600 Wh kg^−1^), non-flammable aqueous electrolytes, environmental benignity, and low cost (∼$3 per kg of zinc).^9–11^ The global ZAB market, valued at about $2.35 billion in 2025, is projected to grow rapidly, reflecting increasing interest in both industrial and portable applications. However, the widespread deployment of ZABs is fundamentally limited by the sluggish kinetics of the oxygen reduction (ORR) and oxygen evolution reactions (OER) at the air cathode.^12^ Hence, the design of efficient, durable, and affordable bifunctional electrocatalysts capable of simultaneously promoting ORR and OER is crucial for realizing the full potential of ZAB technology.^13,14^

Conventional noble metal-based catalysts, such as Pt for ORR and RuO_2_/IrO_2_ for OER, exhibit superior intrinsic activity but suffer from high cost, scarcity, and poor long-term durability under reversible oxygen reaction conditions.^15^ To overcome these limitations, extensive efforts have been devoted to develop earth-abundant transition metal-based catalysts, among which spinel ferrites have attracted particular attention owing to their rich redox chemistry, tunable electronic structures, and intrinsic catalytic activity.^16,17^ Specifically, cobalt ferrite (CoFe_2_O_4_) stands out as a promising candidate due to its high theoretical capacity (∼916 mAh g^−1^), high oxidation-state activity of cobalt with the structural stability of iron, and abundant active sites.^18^ However, pristine CoFe_2_O_4_ suffers from poor electrical conductivity and degradation caused by volume expansion during cycling, which limits its electrochemical performance.^19,20^ To address these challenges, integrating transition metal oxides with conductive carbon nanostructures such as carbon nanotubes (CNTs), graphene, or carbon nanofibers (CNFs) has proven effective.^21,22^ Carbon materials enhance electron transport, prevent nanoparticle aggregation, improve mechanical robustness, and modulate the electronic structure of the active sites, thereby accelerating ORR and OER.^23,24^ In particular, electrospun porous carbon nanofibers (PCNFs) due to their high surface area, tunable porosity, interconnected architecture, and scalability, promote ion/electron diffusion and mechanical stability during cycling.^25^ Alshehri *et al.* reported that CoFe_2_O_4_ nanoparticles embedded in nitrogen-doped mesoporous carbon exhibited excellent stability, with overpotentials of 164 mV (HER) and 370 mV (OER) at 10 mA cm^−2^, and a cathodic ORR peak at 0.85 V.^26^ Likewise, L. Wang, *et al.* reported that nano-sized CoFe_2_O_4_ particles were uniformly deposited on the surface of porous CNTs and tested the material as anode for lithium-ion battery where it showed exceptional electrochemical performance with high lithium-storage capacity of 1077 mAh g^−1^ after 100 cycles, and rate capability (694 mAh g^−1^ at 3 A g^−1^).^27^

A persistent challenge, however, lies in achieving uniform dispersion and strong interfacial bonding between metal oxide nanoparticles and carbon supports. Conventional methods often yield heterogeneous particle sizes and weak interfaces, leading to agglomeration and poor stability. Surface functionalization using catechol-containing molecules, such as dopamine and its polymerized derivative polydopamine, has proven highly effective in anchoring metal species through coordination bonding, π–π interactions, and electrostatic attraction.^28,29^ However, in many reported systems, dopamine/catechol functionalization has mainly been used as an adhesive or surface modification layer, while its role as a structure-directing interface for regulating spinel ferrite growth remains less explored. Taking advantage of the strong metal-ion chelating ability of the catechol groups in dopamine,^30^ this work presents a rational strategy for anchoring ultrafine cobalt ferrite (CoFe_2_O_4_) nanorods on dopamine-functionalized porous carbon nanofibers (PCNFs), denoted as CoFe_2_O_4_@DP@PCNF. This architecture maximizes the exposure of active sites, enhances electronic conductivity, and may facilitates electron transport pathways due to homogenous dispersion and stronger oxide–metal interfacial contact. As demonstrated herein, the catalyst exhibits competent bifunctional activity for both OER and ORR, along with good electrochemical stability and durability, making it a promising candidate for next-generation zinc–air batteries.

## Experimental methods

### Materials and chemicals

Polyacrylonitrile (PAN, ∼*M*_w_ 150 000), polypyrrolidone (PVP, ∼*M*_w_ 40 000), dimethylformamide (DMF), cobalt nitrate hexahydrate (≥98%), urea, ammonium fluoride (≥98%), *N*-hydroxysuccinimde (98%), *N*,*N*′-dicyclohexylcarbodiimide (DCC, 99%), dopamine (≥98%), potassium hydroxide (≥85%) pellets were purchased from Sigma-Aldrich. Iron chloride nanohydrate (≥98%) was purchased from Daejung.

### Synthesis and functionalization of PCNF

A 9 wt% PAN and 1 wt% PVP solution in DMF was electrospun at 20 kV using a needle-to-collector distance of 20 cm and flow rate of 0.5 mL h^−1^. The obtained nanofibers were air-stabilized for 2 h at 280 °C in a muffle furnace using a ramp rate of 2 °C min^−1^, and then the nanofibers were moved to a tube furnace for carbonization at 800 °C for 1 h under nitrogen at a heating rate of 5 °C min^−1^ to yield PCNF.

To introduce carbonyl and hydroxyl groups on the surface of PCNF, 100 grams of PCNF were dispersed in 50 mL of 35 wt% of H_2_O_2_ and stirred for 24 h. Vacuum filtration was used to collect the acid-functionalized PCNFs (A-PCNFs), which were then thoroughly cleaned with deionised water (DI) until the filtrate's pH became neutral. The A-PCNFs were dried for 24 h at 60 °C in a vacuum oven. Then, in a 50 mL Schlenk flask, 80 mg of A-PCNFs were dissolved in 4 mL of anhydrous DMF. After 5 min of nitrogen flushing, the Schlenk flask was sealed with a rubber septum. The Schlenk flask was filled with *N*-hydroxysuccinimide (188 mg in 0.5 mL DMF) and *N*,*N*′-dicyclohexylcarbodiimide (336 mg in 0.5 mL DMF). The mixture was then left to stir for 6 h and ester functionalised CNFs (E-PCNF) were collected after vacuum filtration. To remove unreacted DCC and hydroxysuccinimide, the E-PCNF were washed with distilled ethanol. The product was dried at 60 °C in a vacuum oven for 12 h. This was followed by the addition of 0.8 g of dopamine and 70 mg of ECNF in 5 mL of anhydrous DMF and stirred for 6 h in an inert atmosphere. This introduces catechol functional groups that serve as pinning sites for covalent modification and anchoring sites for transition-metal loading (*e.g.*, Co and Fe). After diluting the mixture with distilled ethanol, these functionalised PCNFs were produced by vacuum filtration using a PVDF membrane filter. The mixture was then dried for 24 h at 60 °C in a vacuum oven. In parallel, another batch of PCNF was treated with 50 mL of 35 wt% H_2_O_2_ under stirring for 24 h to achieve surface activation and is hereafter denoted as unfunctionalized PCNF.

### Cobalt ferrite growth on the surface of functionalized PCNF

The cobalt ferrite was grown on the surface of the obtained functionalized PCNFs through hydrothermal process. 5 mL of anhydrous ethanol was added to 20 mL of DI water and stirred continuously at 500 rpm. Then 2 mL of 1 M Co(NO_3_)_2_·6H_2_O aqueous solution, and 6 mL of 1 M CO(NH_2_)_2_ aqueous solution were gradually added at intervals of ∼1–2 min. This solution was then added with 74.08 mg of NH_4_F and 397 mg of FeCl_2_·9H_2_O, respectively. The solution gradually changed color from light pink to orange. The resulting mixture showed a noticeable colour change from light pink to orange. After 20 min of continuous stirring, the final mixture was transferred into a 45 mL stainless-steel autoclave lined with polytetrafluoroethylene (Teflon). In the Teflon holder, 80 mg of functionalised PCNF were also added to the mixed solution. The autoclave was then heated in an oven for 10 h at 120 °C. The brown Co–Fe bimetallic hydroxide carbonate salt precursors that were grown directly on functionalised PCNF were obtained when it was allowed to spontaneously cool to room temperature in air. The solid product was separated by centrifugation at 1000 rpm for 10 min. The precipitate was washed repeatedly with ethanol and DI water until the supernatant became clear, then vacuum-filtered and dried overnight at 60 °C. This sample was then placed in a quartz tube and calcined at 350 °C for 2 h under an air atmosphere at a heating rate of 5 °C min^−1^ to produce cobalt ferrites over PCNFs *i.e.*, CoFe_2_O_4_@DP@PCNF. The same hydrothermal procedure was employed to synthesize cobalt ferrite on unfunctionalized PCNF (CoFe_2_O_4_@PCNF). For comparison, pristine CoFe_2_O_4_ was also prepared under identical conditions without the addition of PCNF. These samples were used as controls in this study.

### Material characterization

Fourier transform infrared (FTIR) spectra obtained by Bruker Alpha FTIR spectrometer, with a spectral resolution of 1 cm^−1^, was used to confirm the functionalisation of PCNF and activation in case of unfunctionalized PCNF. Powder X-ray diffraction (PXRD) data was collected using a Cu Kα X-ray source (Bruker D2 Phaser) in the 2*θ* scan range of 10°–80°. Energy-dispersive X-ray spectroscopy (EDX)-equipped high resolution scanning electron microscopy (FEI 450 Nova NanoSEM) was used to examine the sizes, shapes, and elemental analysis of the produced catalysts. Using an accelerating voltage of 200 kV, transmission electron microscopy (FEI Tecnai G2 F20) was used to do a more thorough structural analysis. X-ray photoelectron spectroscopy (Thermo Scientific K-Alpha) was used to characterise the chemical states and binding energies and the CasaXPS software was used for the peak deconvolution and analysis. The surface area and pore size distribution studies were performed by N_2_ physisorption using the Quantachrome Nova 2200e equipment (Brunauer–Emmett–Teller).

### Electrochemical characterization

The electrochemical characterizations were done on CHI 730e potentiostat. For OER measurements, the working electrode of glassy carbon, reference electrode of Hg/HgO, and the counter electrode of Pt wire was used in 1 M KOH electrolyte. 2 mg of the sample were finely ground and ultrasonically dispersed in 120 mL of DMF and 10 µl of Nafion solution to make the catalyst ink, which was supported on the working electrode. 5 µl of this slurry was dropped cast on the surface of glassy carbon and allowed to air dry. In all electrochemical measurements, the reference electrode was calibrated to the reversible hydrogen electrode (RHE) based on the equation *E*_RHE_ = *E*_(Hg/HgO)_ + (0.059 × pH) + 0.098 V.^31^ Linear sweep voltammetry (LSV) with a scan rate of 5.0 mV s^−1^ was conducted for all catalysts. Electrochemical impedance spectroscopy (EIS) measurements were performed in the frequency range from 10^6^ to 0.1 Hz. Cyclic voltammetry (CV) measurements were used to determine the double layer capacitance *C*_dl_ at several scan rates between 50 and 500 mV s^−1^. At 1.12 V *vs.* RHE, the anodic (*j*_a_) and cathodic (*j*_c_) current densities were measured and displayed as (*j*_a_ − *j*_c_). In the non-faradaic zone, the specific capacitance *C*_s_ was 0.015 mF cm^−2^ based on literature reports for similar carbon-based electrodes.^32^ The electrochemically active surface area (ECSA) is determined by the *C*_dl_/*C*_s_ value, which should be interpreted as relative indicator of electrochemically accessible surface. Long-term stability was assessed by chronoamperometry at a potential of 1.59 V *vs.* RHE for 48 h. Likewise, a three-electrode system was used to do the ORR measurements: a reference electrode of Hg/HgO, a counter electrode of platinum wire, and a working electrode of glassy carbon rotating disc electrode (RDE) (GC, with disk area 0.196 cm^2^). ORR was also conducted in 1 M KOH to maintain consistency between ORR and OER measurements, enabling a reliable evaluation of the bifunctional catalytic performance under identical experimental conditions relevant to Zn–air battery testing. To saturate the electrolyte of 1 M KOH, O_2_ (or Ar) was bubbled through it for 30 min. Prior to recording the CV in an O_2_ saturated electrolyte (scan rate 10 mV s^−1^), the working electrode was cycled 25 times (scan rate 100 mV s^−1^) under Ar gas purging. Linear sweep voltammetry (LSV) was carried out between 225 and 2500 rpm. To estimate the electron transfer numbers in oxygen reduction reaction (ORR), Koutecky–Levich (K–L) plots, using the below equations,^33^ were used
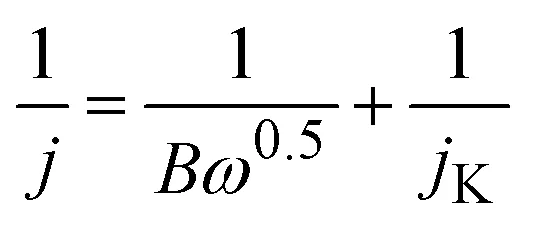
*B* = 0.2*nF*(*D*_O_2__)^2/3^*v*^−1/6^*C*_O_2__where *j* is the obtained electron current density, *j*_K_ is the kinetic current density, *ω* is the rpm of the working electrode, *n* is the electron transfer number, *F* is the Faraday constant (96 485 C mol^−1^), *D*_O_2__ is the O_2_ diffusion coefficient (1.9 × 10^−5^ cm^2^ s^−1^), *C*_O_2__ is O_2_ bulk concentration (1.2 × 10^−6^ mol cm^−1^); *ν* is the kinetic viscosity of the electrolyte (0.01 cm^2^ s^−1^).

## Results and discussion

The Scheme 1 below illustrates the typical synthesis process of cobalt ferrite nanorods decorated on catechol functionalized porous carbon nanofibers (CoFe_2_O_4_@DP@PCNF) *via* electrospinning and subsequent thermal treatments. The nanofibers were prepared by electrospinning the precursor polymeric solution followed by stabilization and carbonization. PVP being a good sacrificial polymer degrades around 350 °C and leaves pores inside the carbon nanofiber.^34^ The PCNF were functionalized through a three-step procedure (Fig. 1), where PCNFs were first functionalized with acid using 35% H_2_O_2_. The surface oxidation of PCNFs introduced various oxygen-containing functionalities, including carboxylic (–COOH) groups. The carboxyl groups contain both hydroxyl (–OH) and carbonyl (C=O) functionalities and were subsequently converted to good leaving groups by reacting with *N*-hydroxysuccinimde (NHS) in the presence of *N*,*N*′-dicyclohexylcarbodiimide (DCC). Finally, catechol groups were introduced by using dopamine as a reactant. The IR spectra of the functionalized CNFs confirmed the successful introduction of the targeted chemical groups at each step. While the present synthesis route involves conventional chemical reagents, the long-term stability of the catalyst may help reduce replacement frequency and improve material utilization. Future studies may further explore more sustainable and environmentally benign synthesis approaches.

**Scheme 1:**
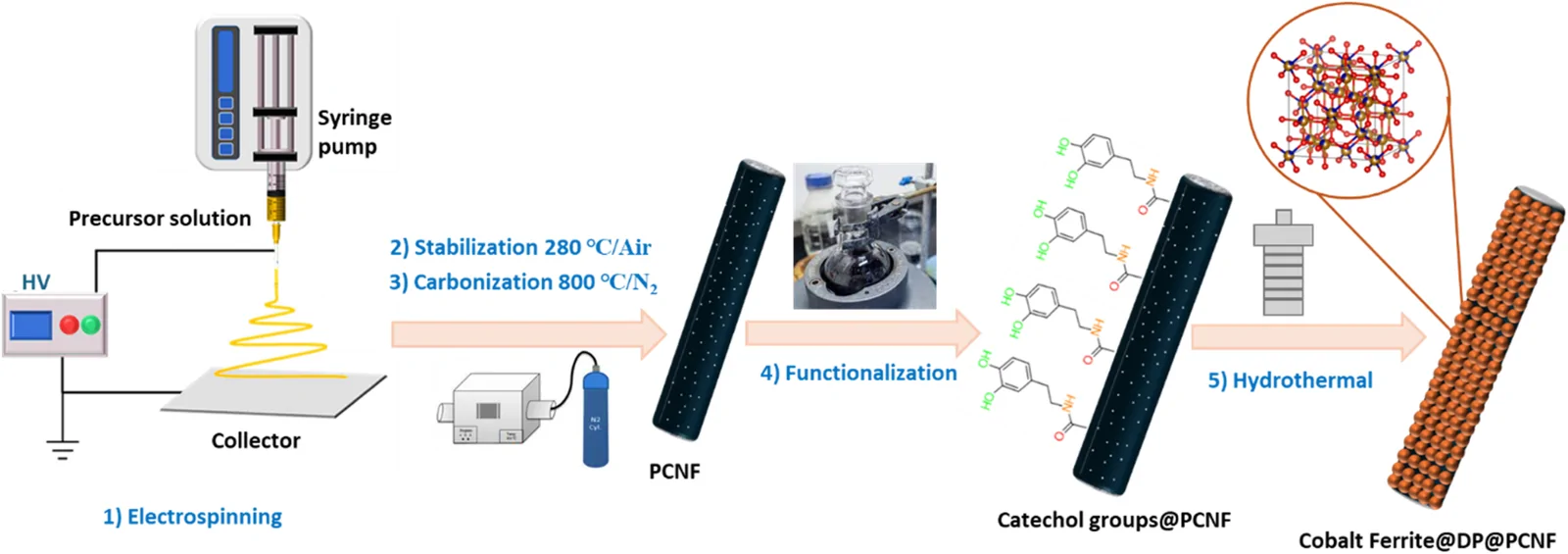
Illustration of the synthesis of CoFe_2_O_4_@DP@PCNF.

**Fig. 1:**
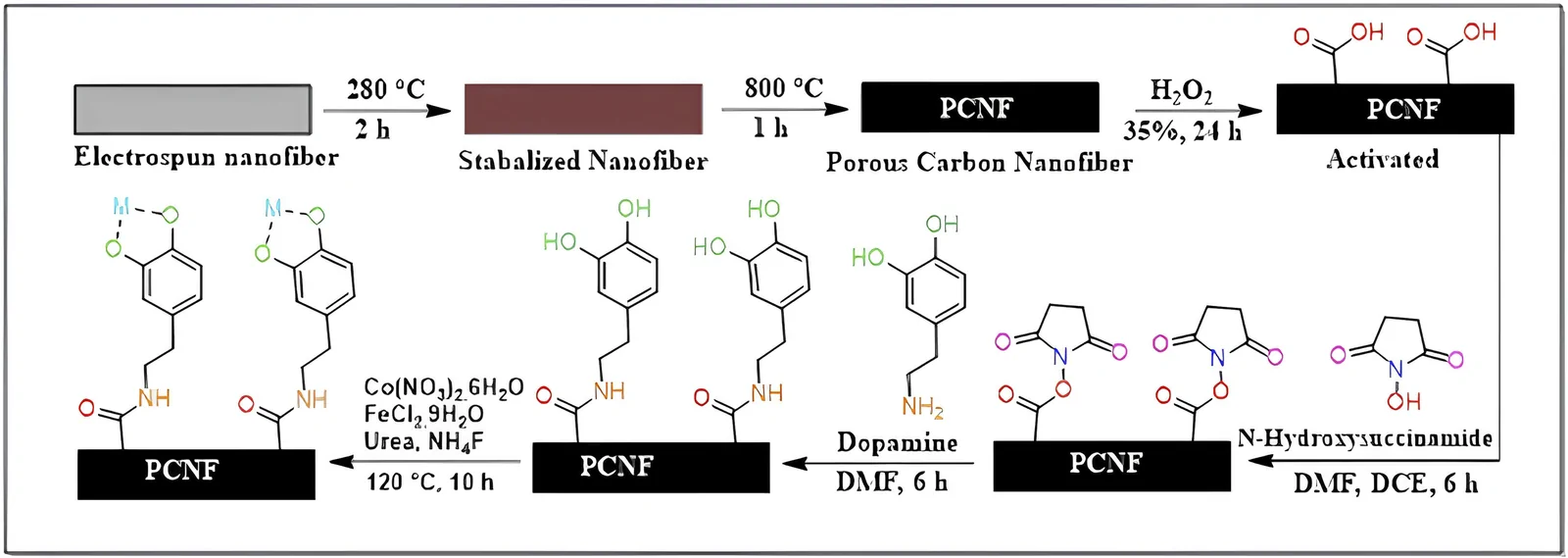
Functionalization steps to induce catechol groups on the surface of PCNF.

In the FTIR spectra shown in the Fig. 2a, pristine PCNF is largely featureless across 4000–500 cm^−1^, consistent with the graphitic carbon bearing few IR-active surface groups. After activation with 35% H_2_O_2_, a distinct broad peak of –OH appears at 3025 cm^−1^ along with carbonyl group peak at 1685 cm^−1^ which confirms the induction of carboxylic acid. Then after further functionalization steps, a distinct band emerges with catechol group that are diagnostic of the grafted moiety: a broad envelope at 3400–3200 cm^−1^ attributable to N–H stretching and –OH group, aliphatic C–H stretches at 2920 and 2850 cm^−1^ from the alkyl spacer, a strong amide-I C=O band centered near ∼1670 cm^−1^, and the amide-II band at ∼1550 cm^−1^ (N–H bending/C–N stretching). Additional features in the 1250–1020 cm^−1^ region arise from the C–O/C–N stretching of the amide/aryl ether domain. These characteristic bands support the introduction of dopamine/catechol-derived functional groups onto the PCNF surface. Aromatic ring modes and out-of-plane C–H bending appear below 900 cm^−1^. The absence of these bands in unfunctionalized PCNF and their concurrent appearance with functionalization confirms the successful covalent attachment of the amide-linked aryl/phenoxide ligand to the PCNF surface. Catechol functionalization is known to interact with transition metal ions such as Fe^3+^, Zn^2+^ and Co^2+^ and is, therefore, likely to influence subsequent metal deposition by providing localized coordination sites, as supported by literature reports.^35,36^ This is expected to promote more uniform nucleation of CoFe_2_O_4_.

**Fig. 2:**
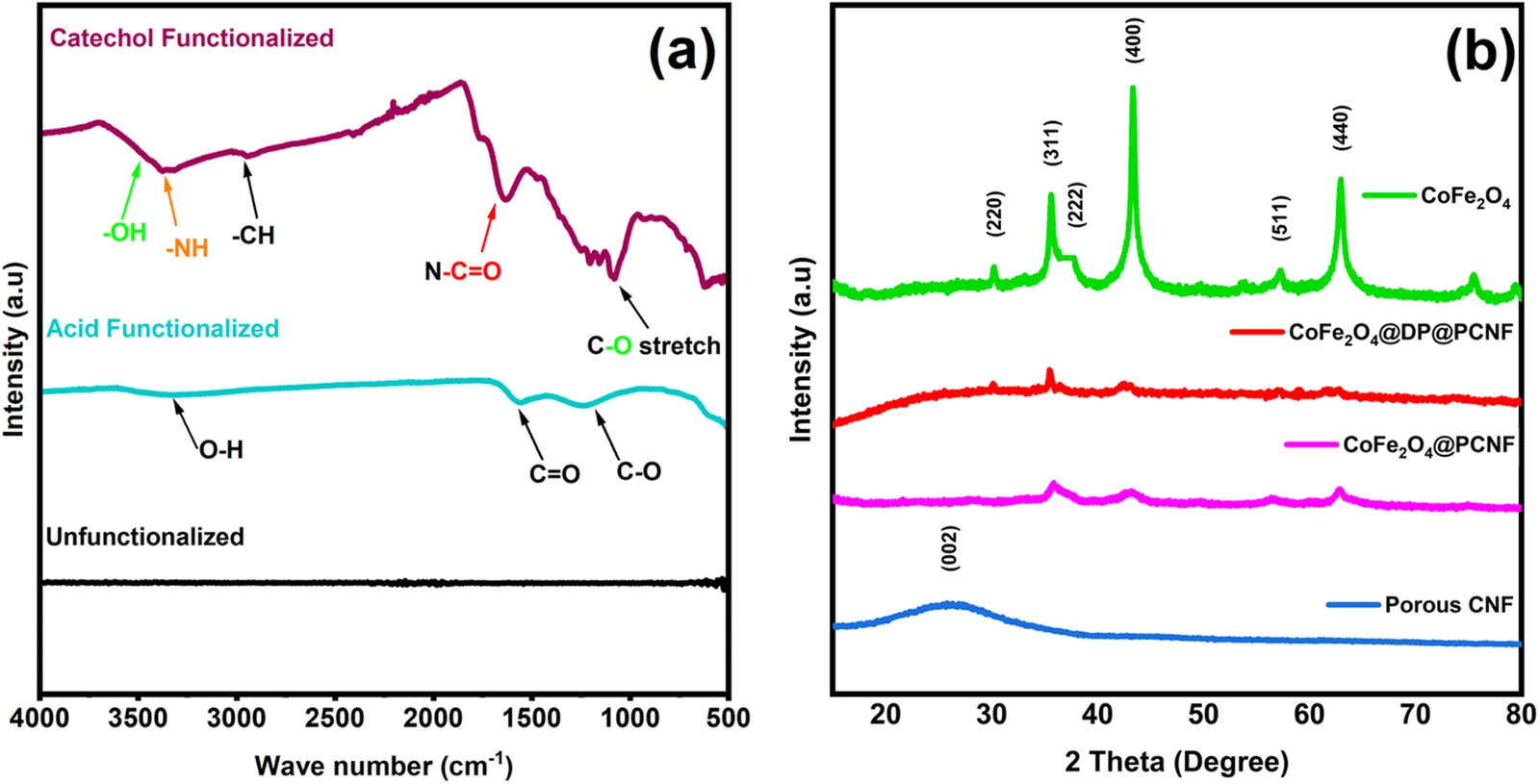
(a) FTIR spectra showing the functional groups introduced on the PCNF surface, and (b) Powder XRD spectra of all the samples.

The XRD scans of the synthesized samples in Fig. 2b confirms the successful formation of CoFe_2_O_4_ in all three cases. The pristine CoFe_2_O_4_ sample exhibits sharp diffraction peaks at 2*θ* values corresponding to the (220), (311), (222), (400), (511), and (440) planes, which are consistent with the standard spinel cubic structure of CoFe_2_O_4_ (JCPDS No. 22-1086).^37^ Similar diffraction peaks are observed for CoFe_2_O_4_@PCNF and CoFe_2_O_4_@DP@PCNF, verifying that CoFe_2_O nanorods are successfully formed and decorated on the surface of the PCNF. The pristine PCNF sample displays a broad peak around 2*θ* ≈ 25°, attributed to the (002) plane of graphitic carbon, indicating its predominantly amorphous nature. The broadened (311) and (222) diffraction peaks of CoFe_2_O_4_@DP@PCNF indicate reduced crystallite size which is consistent with catechol-mediated regulation of nucleation and crystal growth and may also reflect interfacial interactions between CoFe_2_O_4_ and the amorphous carbon framework. The smaller crystallite size is expected increase the accessible active sites and boost electrolytic performance of the catalyst.^38,39^

The surface morphology of cobalt ferrite nanorods deposited on PCNF was investigated using scanning electron microscopy (SEM). In case of CoFe_2_O_4_@PCNF, the nanorods exhibited an average size of ∼103 ± 4 nm in length and were uniformly distributed across the nanofiber surface (Fig. 3a–c). While upon functionalization of PCNF with dopamine, a significant reduction in nanorod size *via* catechol groups was achieved, with nanorods exhibiting an average length of ∼29 ± 1.5 nm, as confirmed by the size distribution analysis (Fig. S1). Notably, the smaller nanorods remained homogeneously distributed along the nanofiber framework (Fig. 3d–f). The observed reduction in size can be attributed to the catechol groups of dopamine, which provide abundant anchoring sites, increase nucleation density, and suppress nanorod overgrowth. This controlled deposition process yielded a finer dispersion and a more stable nanostructured coating compared to the non-functionalized PCNF system.

**Fig. 3:**
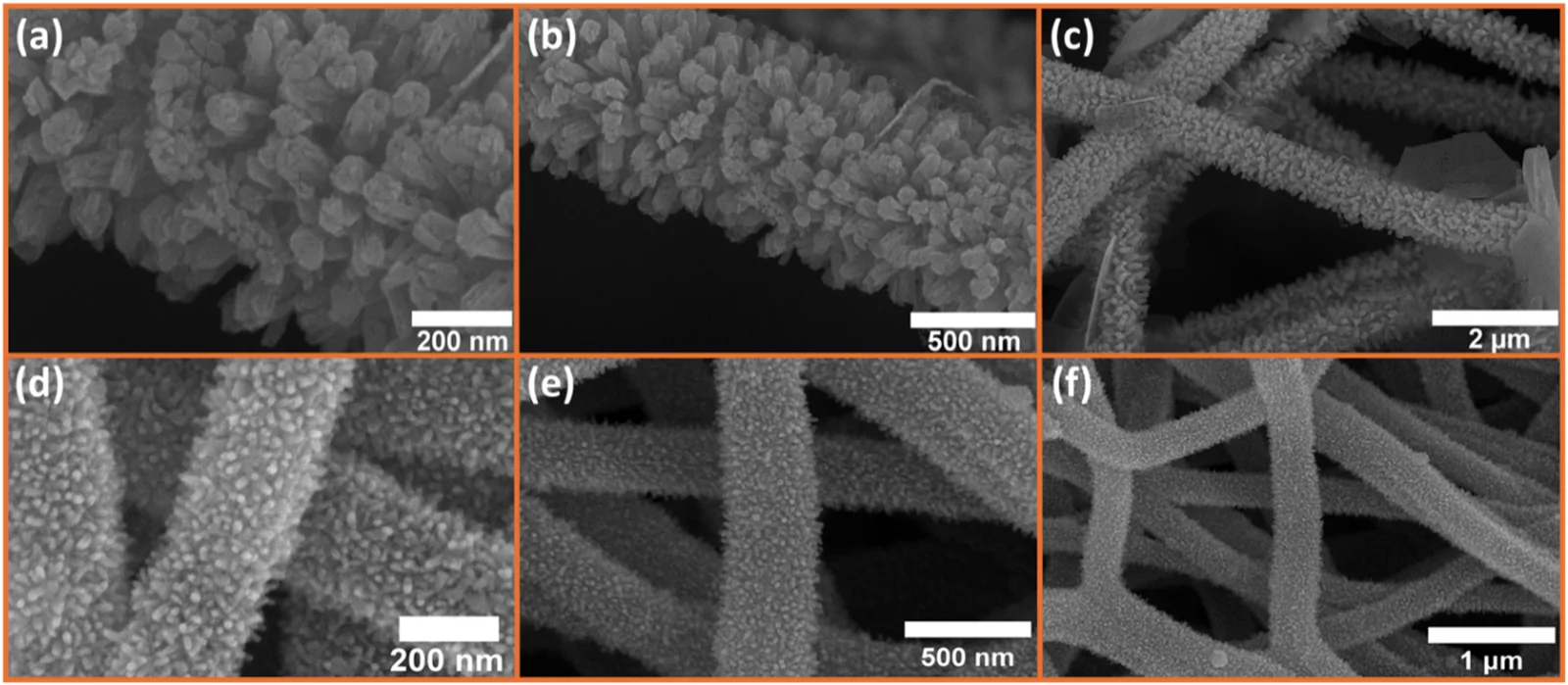
SEM images of (a)–(c) CoFe_2_O_4_@PCNF and (d)–(f) CoFe_2_O_4_@DP@PCNF.

To further investigate the internal structure and elemental composition of the sample, the transmission electron microscopy (TEM), energy dispersive X-ray spectroscopy (EDX) and elemental mapping were performed. The TEM image in Fig. 4a reveals a porous morphology and shows that the CoFe_2_O_4_ nanorods are uniformly distributed on functionalized PCNF with sizes in the range of ∼29 ± 1.5 nm, consistent with the SEM observations. The low-magnification TEM image (Fig. 4b) further illustrated that the fibrous network retained its structural integrity and was homogeneously decorated with nanorods across the interconnected framework. The high-resolution TEM in Fig. 4c revealed well-defined lattice fringes with an interplanar spacing of 0.26 nm, corresponding to the characteristic (311) crystallographic plane of spinel CoFe_2_O_4_, while the inset SAED pattern confirmed the crystalline nature of the nanorods. The EDX elemental maps captured in SEM (Fig. 4d–g) further show homogeneous distribution of elements.

**Fig. 4:**
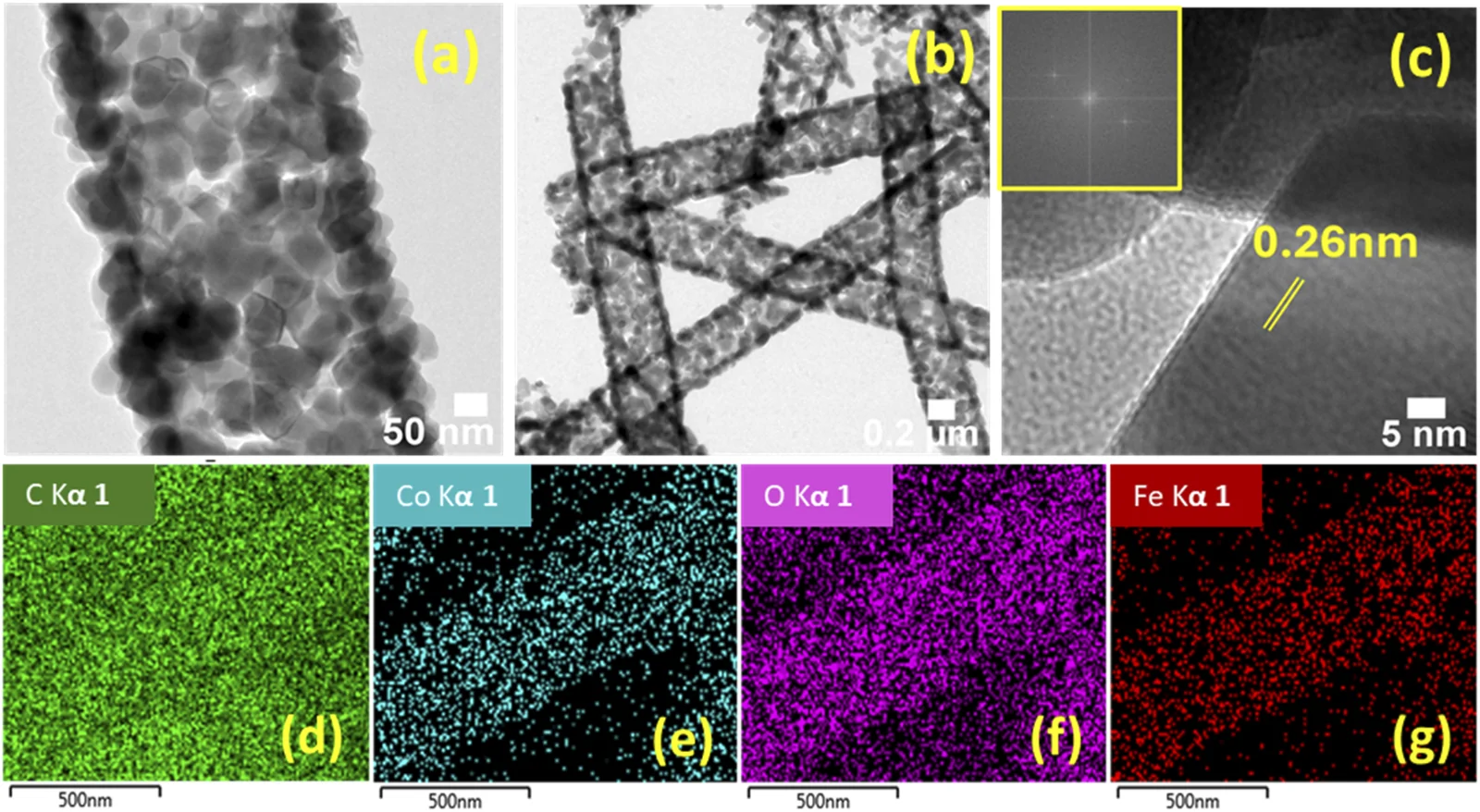
(a) and (b) TEM images, (c) HRTEM image, and (d)–(g) SEM-EDX elemental maps of CoFe_2_O_4_@DP@PCNF.

Fig. S3(a)–(c) presents the SEM image of cobalt ferrite synthesized without any support, where clear agglomeration of nanorods is observed. This highlights the critical role of carbon nanofibers as an effective support material in preventing aggregation. In contrast, Fig. S4(a)–(c) shows the SEM image of PCNF, displaying a smooth and fine morphology, and the associated EDX spectrum reveals the exclusive presence of carbon without detectable impurities, further validating the purity of the nanofiber support.

The N_2_ adsorption–desorption isotherm (Fig. S5a) exhibits a clear type-IV curve with a pronounced hysteresis loop, indicating the mesoporous nature of CoFe_2_O_4_@DP@PCNF. The significant increase in nitrogen uptake at low relative pressures and the well-defined capillary condensation region indicate the presence of accessible and interconnected pores. The shape of the hysteresis loop depicts that the catalyst has wedge-like pores.^40^ The catalyst exhibits a BET specific surface area of 51.77 m^2^ g^−1^ (*R*^2^ = 0.9999), confirming reliable surface area determination. Additionally, pore size distribution graph (Fig. S5b) confirmed a dominant pore size around 3.8 nm with a total pore volume of 0.182 cm^3^ g^−1^, consistent with the mesoporous behavior observed in the isotherm.

X-ray photoelectron spectroscopy (XPS) was used to acquire further understanding of the surface composition and oxidation states. Fig. 5a shows the survey spectrum of CoFe_2_O_4_@DP@PCNF that clearly indicates the presence of Co 2p, Fe 2p, O 1s, N 1s, and C 1s peaks, at binding energies (BE) of 784.16, 714.71, 533.01, 399.13, and 284.10 eV, respectively. XPS fitting was performed using Shirley background subtraction after calibration to C 1s at 284.8 eV. All component spectra were fitted using literature-guided binding-energy positions, spin–orbit splitting, FWHM constraints, and satellite features to minimize overfitting and ensure physically meaningful oxidation-state assignment. The C 1s spectrum Fig. 5b shows a prevalent peak at ∼284.6 eV, assigned to graphitic C=C from the PCNF backbone. A component at ∼285.8 eV corresponds to C–N species, while the peak at ∼288.5 eV is identified to O–C=O groups from carboxylate functionalities. The O 1s spectrum of cobalt ferrite shown in Fig. 5c is deconvoluted into three components, a dominant peak at ∼530.0 eV corresponding to lattice oxygen (O^2−^ in Co–O/Fe–O bonds) confirming the presence of the spinel oxide phase, a higher binding energy feature at ∼531.5 eV attributed to surface hydroxyl groups, and a minor component at ∼533.2 eV arising from adsorbed oxygenated species, consistent with carboxylate or C–O containing groups on the surface of PCNF. The deconvoluted N 1s spectra presented in Fig. 5d displays two peaks at 395.10 and 397.80 eV, which are the signature peaks of the pyridinic nitrogen and amide group nitrogen (N–C=O) induced due to functionalization, respectively. The XPS spectra of Fe 2p (Fig. 5e) exhibits two large fitting peaks at the binding energy of 710.5 and 723.9 eV, along with two small satellite peaks at 718.4 and 733.7 eV, which correspond to Fe 2p_3/2_ and Fe 2p_1/2_, respectively. The presence of Fe^3+^ and Fe^2+^ in the octahedral and tetrahedral sites of inverse spinel is primarily attributed to the deconvolution spin–orbit spectrum peaks of the Fe 2p_3/2_ and Fe 2p_1/2_. Similarly, the XPS spectrum of the Co 2p (Fig. 5f) contains two significant peaks related to Co 2p_3/2_ and Co 2p_1/2_, with the weak satellite peaks at binding energies of 787.7 eV and 803.2 eV. These two shake-up peaks of Co at the high binding energy side of the Co 2p_3/2_ and Co 2p_1/2_ edge show the presence of Co^2+^ in the sample. The peaks are centred at 780.4 and 795.6 eV. The separation of satellite peaks and the peak widths of the Co 2p_3/2_ and Co 2p_1/2_ signals suggest that Co^2+^ and Co^3+^ co-exist in the CoFe_2_O_4_@DP@PCNF. The XPS of all other samples CoFe_2_O_4_@PCNF, CoFe_2_O_4_, PCNF are given in SI Fig. S6–S8.

**Fig. 5:**
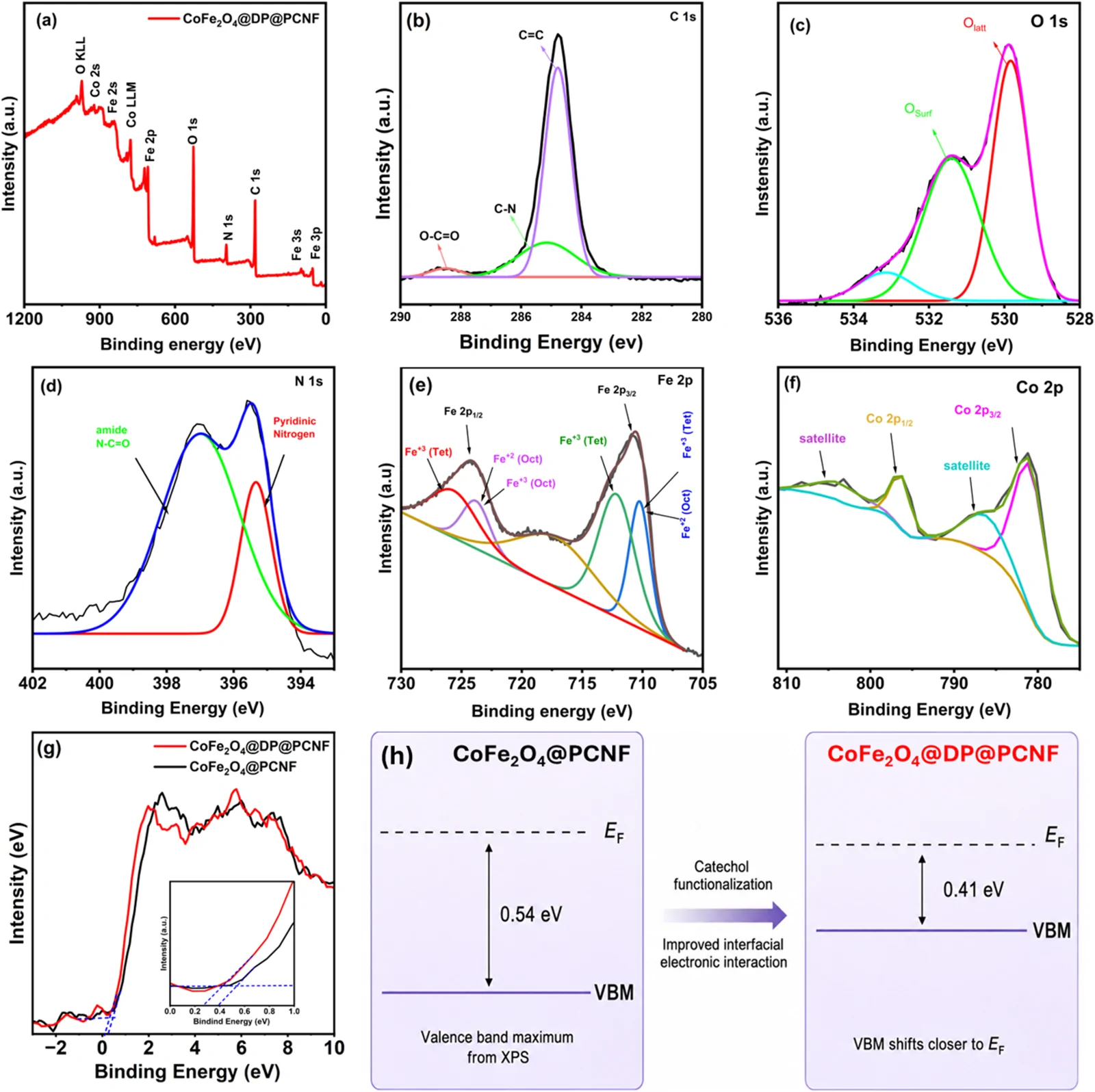
XPS analysis of CoFe_2_O_4_@DP@PCNF. (a) Survey scan, and high-res (b) C 1s, (c) O 1s, (d) N 1s, and (e) Fe 2p, and (f) Co 2p scans. (g) Valance-band XPS of CoFe_2_O_4_@CNF and CoFe_2_O_4_@DP@CNF and (h) schematic of the valence-band alignment.

The coexistence of Co^2+^/Co^3+^ and Fe^2+^/Fe^3+^ observed in the high-resolution XPS analysis is consistent with the mixed-valence nature of the inverse spinel structures. Therefore, these oxidation states alone cannot be considered direct evidence of enhanced catalytic activity. To further examine whether catechol functionalization modulates the electronic structure of the ferrite phase, valence band XPS analysis was performed as shown in Fig. 5g and h. The valence band maximum shifted from 0.54 eV for CoFe_2_O_4_@PCNF to 0.41 eV for CoFe_2_O_4_@DP@PCNF, indicating that the valence band edge moves closer to the Fermi level after catechol functionalization. This shift may suggest electronic modulation at the ferrite–carbon interface and a reduced energy barrier for electron transfer, which can facilitate charge transport during electrocatalysis.

## Oxygen evolution reaction (OER)

A standard three-electrode system with inert glassy carbon as working electrode was utilized to determine the catalytic performance of all samples for OER in alkaline medium (pH = 13.85). Fig. 6a represents the LSV curves, where CoFe_2_O_4_@DP@PCNF shows competent OER performance with an overpotential of 301 ± 4 mV to drive 10 mA cm^−2^, surpassing the benchmark RuO_2_, which required an overpotential of 410 mV to achieve this current density.^41^ In comparison, CoFe_2_O_4_@PCNF, pristine CoFe_2_O_4_, DP@PCNF and PCNF required higher overpotentials of 400 ± 3 mV, 580 ± 7 mV, 650 ± 3 and 670 ± 2 mV respectively. The corresponding Tafel slopes for all the catalyst are shown in Fig. 6b. The faster kinetics and lower overpotential in case of CoFe_2_O_4_@DP@PCNF again demonstrates the beneficial role of catechol functionalization in tailoring the structural and enhancing the interaction of CoFe_2_O_4_ with the carbon support. The catechol moieties act as anchoring and coordinating agents,^42^ improving dispersion, conductivity, and active-site exposure, thus, playing an important role in lowering the kinetic barrier for OER.^43^ Hence, enhanced catalytic activity is likely associated with the synergistic effects of catechol functionalization, including morphology regulation, improved homogeneity of nanorods, surface exposure, and modulation of interfacial/electronic interactions. It is also worth noting that CoFe_2_O_4_ samples without PCNF support show the poorest OER performance. Because the porous carbon support offers a conductive channel and a greater defect structure for the adsorption and desorption of intermediates, it accelerates electron transfer and improves OER performance, which is better than recently reported for similar materials. For example, N-doped graphene quantum dots incorporating cobalt ferrite/graphitic carbon nitride,^44^ CoFe alloy/N-doped carbon^45^ and FeCo encapsulated in N-doped graphene^46^ required overpotential of 445 mV, 369 mV and 440 mV overpotential to reach a current density of 10 mA cm^−2^ and a Tafel slope of 69 mV dec^−1^, 77 mV dec^−1^ and 90 mV dec^−1^ respectively. The performance of CoFe_2_O_4_@DP@PCNF catalyst is compared with other reported cobalt-based electrocatalysts in Table S1. Moreover, the charge-transfer characteristics at the electrode–electrolyte interface of CoFe_2_O_4_@DP@PCNF and CoFe_2_O_4_@PCNF were further investigated through the exchange current density (*J*_ex_), which was determined by extrapolating the linear region of the Tafel plot to zero overpotential (Fig. 6b). CoFe_2_O_4_@DP@PCNF exhibited a significantly higher *J*_ex_ of 35 µA cm^−2^ compared to 17 µA cm^−2^ for CoFe_2_O_4_@PCNF, indicating faster interfacial electron-transfer kinetics and enhanced charge transport during the water oxidation reaction.

**Fig. 6:**
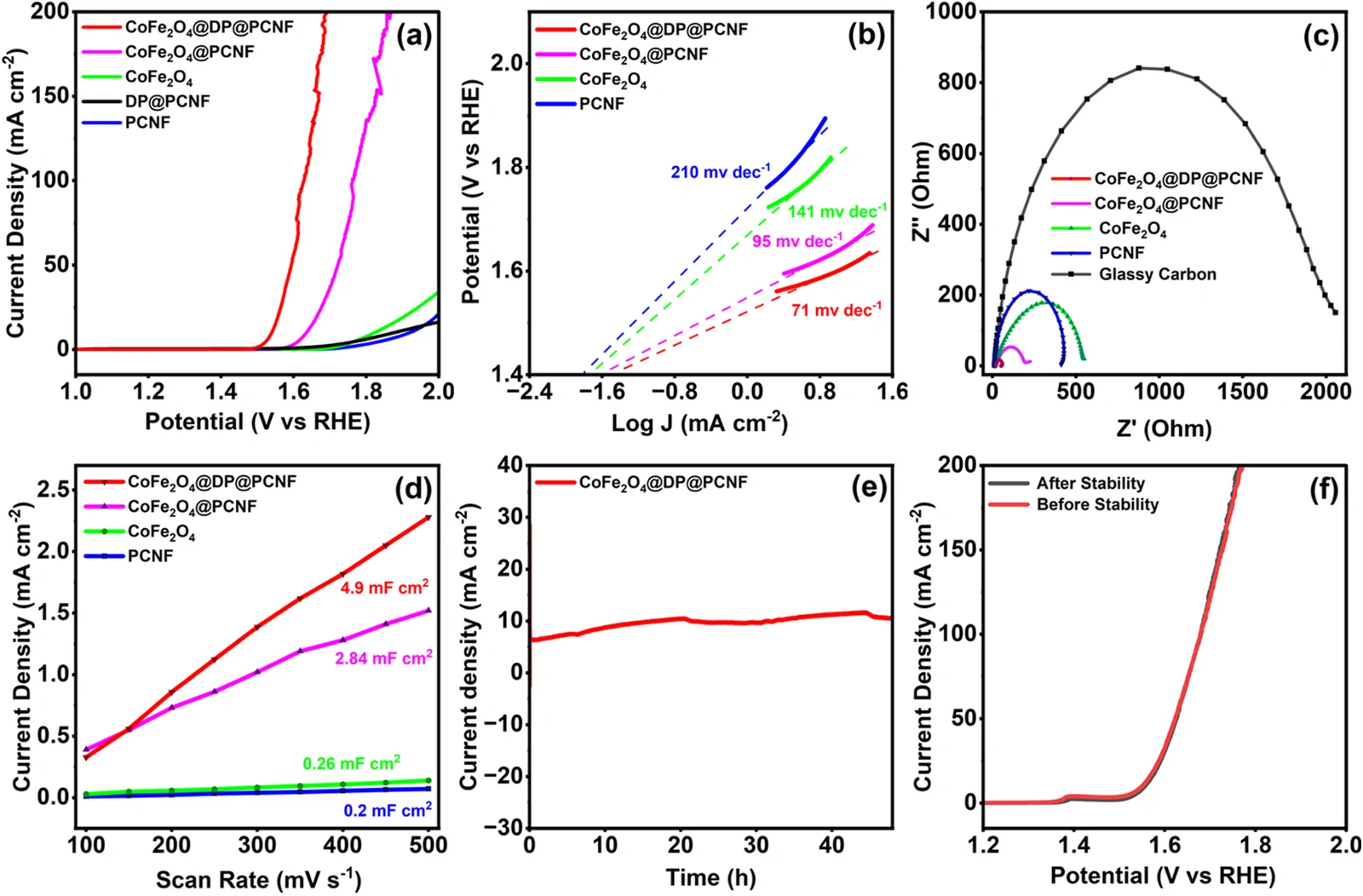
(a) OER polarization curves with 95% *iR*-compensation, (b) Tafel slopes, (c) Nyquist plots recorded in 1.0 M KOH solution, and (d) electrochemical surface area of the catalysts. (e) Chronoamperometric curve at a potential of 1.59 *vs.* RHE under same *iR*-compensation condition for 48 h and (f) OER polarization curve before and after 48 h stability run for CoFe_2_O_4_@DP@PCNF.

Electrochemical impedance spectroscopy revealed that CoFe_2_O_4_@DP@PCNF catalyst faces the least charge transfer resistance of 26.13 Ω, while CoFe_2_O_4_@PCNF, CoFe_2_O_4_ and PCNF showed 54.84, 117.21 and 211.58 Ω, respectively (Fig. 6c). ECSA was estimated for all samples by measuring their double layer capacitance (*C*_dl_) *via* cyclic voltammetry (CV) in the non-faradic region and the results are given in Fig. 6d. Because the cobalt ferrites were grown to a regulated size, the ECSA of CoFe_2_O_4_@DP@PCNF was the highest of all the samples, measuring 326.66 cm^2^, indicating higher exposure of accessible active sites. Fig. S9–S12a, displays the CV at various scan rates in the non-faradic region of all samples, which is utilised to calculate the double layer capacitance in Fig. S9–S12b, for determining the ECSA. To gain deeper insight into the intrinsic electrocatalytic behavior of the catalysts, the LSV curves were normalized with respect to the ECSA and BET surface area using eqn (S1) and (S2). The ECSA-normalized and BET-normalized polarization curves (Fig. S13a and b) showed that CoFe_2_O_4_@DP@PCNF required a lower overpotential to drive the OER process, confirming its competitive intrinsic electrocatalytic activity as well as clear correlation between morphology and electrocatalytic performance.

The OER stability of CoFe_2_O_4_@DP@PCNF was evaluated by chronoamperometry at a constant potential of 1.59 V *vs.* RHE (Fig. 6e). The catalyst showed good stability by maintaining its activity even after 48 h. This is one of the best reported activities for OER on glassy carbon electrode (GCE) under steady state conditions. Fig. 6f shows that the polarization curves overlap even after 48 h with negligible shift of ∼3 mV which signifies that CoFe_2_O_4_@DP@PCNF can be used as a highly competent electrocatalyst for OER with much better durability for long-term use. A similar reaction was carried out in a H-cell setup where the oxygen produced during the reaction was collected and quantified using the water displacement method as shown in Fig. S14. The faradaic efficiency for the generated oxygen was calculated using the eqn (S3) and (S4) and found to be 98.47%. Furthermore, the average intrinsic activity of the individual active sites was estimated by calculating their turnover frequencies (TOFs) using eqn (S5) based on the electrochemical metal redox peak integration of each sample (Fig. S15). At a potential of 1.62 V, CoFe_2_O_4_@DP@PCNF, CoFe_2_O_4_@PCNF, and pristine CoFe_2_O_4_ delivered TOF values of 0.221, 0.143, and 0.072 O_2_ s^−1^ (Fig. S16), respectively. In accordance with the normalized LSVs, the substantially higher TOF observed for CoFe_2_O_4_@DP@PCNF, nearly twice that of the CoFe_2_O_4_@PCNF, demonstrates improved accessibility and utilization of catalytically active sites. To gain deeper mechanistic insight into the enhanced OER activity, the apparent activation energy (*E*_a_) was determined to evaluate the reaction kinetics of the catalysts. The *E*_a_ values were calculated according to the Arrhenius relationship (eqn (S6)), and the integrated form (eqn (S7)). LSV measurements were performed at different temperatures *i.e.* 20 °C, 40 °C and 60 °C, and the activation energies were extracted from the slopes of the Arrhenius plots (Fig. S17) which exhibited very good linearity, with *R*^2^ values of 0.99 and 0.98 for the catechol- and carboxyl-functionalized samples, respectively, confirming that the experimental data are well described by the Arrhenius relationship. Notably, CoFe_2_O_4_@DP@PCNF exhibited a significantly lower *E*_a_ value of 20.06 kJ mol^−1^ at 1.55 V *versus* RHE compared with 28.29 kJ mol^−1^ for CoFe_2_O_4_@PCNF, indicating substantially accelerated OER kinetics (Fig. S18). The reduced activation barrier suggests that the catechol-functionalized PCNF support effectively regulates the nucleation and growth of CoFe_2_O_4_ nanoparticles, enhancing the exposure of catalytically active sites and promoting interfacial charge-transfer. Consequently, less energy is required to drive the OER on CoFe_2_O_4_@DP@PCNF. Furthermore, a gradual decrease in *E*_a_ with increasing applied potential was observed for both catalysts, implying that the reaction pathway remains kinetically consistent within the investigated potential range. However, the lowering of *E*_a_ at higher potential is usually expected in electrolytic system as it stabilizes the transition state associated with the rate-determining step, thereby reducing the overall energy barrier.^47,48^ Consequently, the catalyst exhibits a higher TOF, lower activation energy, and good OER performance, establishing a clear structure–activity relationship between morphology and catalytic activity.

## Oxygen reduction reaction (ORR)

Cyclic voltammetry (CV) and linear sweep voltammetry (LSV) were taken at a scan rate of 10 mV s^−1^ in N_2_ and O_2_ saturated 1 M KOH solution (pH = 13.85). The CV plots for the CoFe_2_O_4_@DP@PCNF catalyst (Fig. 7a) showed no peaks in the N_2_ saturated environment (dashed data) but a significant oxygen reduction peak at 0.85 V *vs.* RHE in O_2_ saturated electrolyte. Under similar testing conditions, the other catalyst CoFe_2_O_4_@PCNF, CoFe_2_O_4_ and PCNF exhibits ORR peak around 0.79 V, 0.67V and 0.54V *vs.* RHE. Fig. 7b shows the LSV data of all catalysts at 1600 rpm and the onset potential (*E*_onset_), half-wave potential (*E*_1/2_), and current densities (*J*_0.4V_) acquired from the LSV measurements for all are summarized in Table S2. Among all the catalysts, CoFe_2_O_4_@DP@PCNF showed competent activity with *E*_onset_ and *E*_1/2_ of 0.90 V and 0.77 V *vs.* RHE and a higher limiting current density. It is important to note that the ORR activity was evaluated in 1 M KOH to maintain consistency with the OER testing conditions for bifunctional assessment. Despite the relatively lower oxygen solubility and increased mass-transport resistance in concentrated alkaline electrolyte,^49^ CoFe_2_O_4_@DP@PCNF still exhibited appreciable ORR activity, further supporting its bifunctional catalytic capability. The catalyst shows even better activity than some of the cobalt-based electrocatalysts reported in Table S3 and can be ascribed to the synergistic integration of ultrafine cobalt ferrite nanorods homogeneously anchored on PCNF through catechol functionalization.

**Fig. 7:**
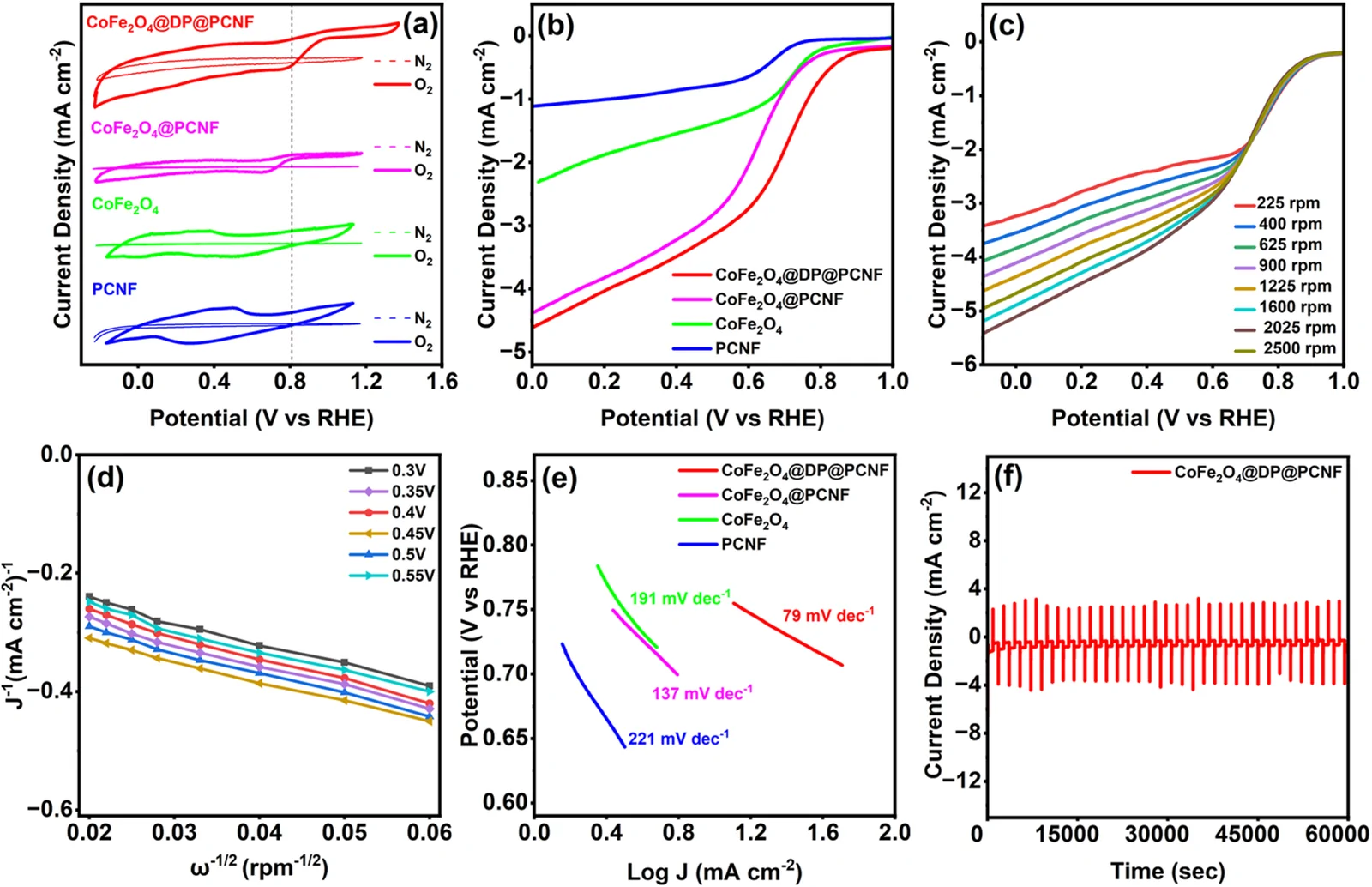
(a) CV plots in O_2_ (solid line) and N_2_ (dashed line) saturated 1 M KOH and (b) LSV polarization curves in O_2_ saturated 1 M KOH at 1600 rpm. (c) LSV at different rpms, (d) corresponding K–L plots, (e) Tafel plots, and (f) stability test of CoFe_2_O_4_@DP@PCNF.

A four-electron (4e^−^) pathway is preferred for achieving the highest ORR efficiency. RDE voltammograms at rotational speeds of 225–2500 rpm is helpful in comprehending the kinetics and 4e^−^ selectivity. Since there is more O_2_ available on the electrode surface at larger rotational speeds, the limiting current density is consequently increased (Fig. 7c). The electron transfer number (*n*) for CoFe_2_O_4_@DP@CNF, estimated from the Koutecky–Levich (K–L) plots, is approximately 4 (Fig. 7d). The Tafel slopes (Fig. 7e) were 79 mV dec^−1^ (CoFe_2_O_4_@DP@CNF), 137 mV dec^−1^ (CoFe_2_O_4_@CNF), 191 mV dec^−1^ (CoFe_2_O_4_), and 221 mV dec^−1^ (PCNF), respectively. The lower Tafel slope for CoFe_2_O_4_@DP@CNF compared to rest of the samples further signify that controlling the size of ferrite improves the catalytic activities on the heterostructure and facilitate charge transport efficiently. The stability of CoFe_2_O_4_@DP@CNF was evaluated in 1 M KOH using an O_2_-saturated electrolyte under 1600 rpm. During the test, a continuous potential-step sequence between 1.0 V and 0.7 V *vs.* RHE was applied for 60 000 seconds instead of a constant-potential chronoamperometry at 0.7 V *vs.* RHE. The corresponding current–time curve (Fig. 7f) showed consistent and reproducible transients throughout the entire experiment, with minimal drift in both peak and steady-state current densities. The average current density over the final 10 minutes maintained more than 90% of its initial value, demonstrating remarkable electrochemical stability under repeated potential fluctuations. Since a pulsed potential typically places greater stress on the catalyst electrode interface than a steady potential hold, this result further confirms the durability of the catalyst toward ORR. Although the procedure differed from the intended fixed-potential chronoamperometric test, it still offers meaningful insight into the structural and electrochemical resilience of the material under dynamic conditions. The impressive robustness of CoFe_2_O_4_@DP@CNF can be attributed to its uniform rod size and the functionalization with catechol, which enhances the availability of CoFe active sites. To examine the possible formation of hydrogen peroxide during the prolonged ORR stability test, the electrolyte was collected after the durability experiment and analyzed quantitatively using an iodometric method,^50,51^ which showed negligible H_2_O_2_ formation (Fig. S19). The ICP-OES analysis of the post-stability electrolytes revealed negligible metal leaching for CoFe_2_O_4_@DP@PCNF, with Fe detected at 1.9 ppm and Co below the detection limit, compared with 5.6 ppm Fe leaching from CoFe_2_O_4_@PCNF. Post-stability XRD analysis was performed (Fig. S20) to evaluate the structural integrity of the catalyst after electrochemical testing. The characteristic (311) and (400) diffraction peaks of CoFe_2_O_4_ at 35.71° and 43.06°, respectively, are largely retained after the stability test confirming the structural stability.

## Zn–air battery testing

The bifunctional activity of CoFe_2_O_4_@DP@PCNF for ORR and OER motivated the testing of the material in rechargeable ZAB. For testing in 6 M KOH electrolyte, carbon cloth loaded with the catalyst served as the air cathode and zinc foil as the anode (Fig. 8a). The open circuit voltage (OCV) of ZAB was maintained at 1.372 V, as shown in Fig. 8b, which is comparable to 1.30 V of commercial 20% Pt/C + RuO_2_ reported in literature.^41^ The measured Zn–air battery OCV demonstrates appropriate cell operation and is consistent with the favorable performance of the developed air electrode. The assembled ZAB exhibits low polarization losses and favorable reaction kinetics, delivering a maximum power density of 127.33 mW cm^−2^ (Fig. 8c). It also achieves a high specific capacity of 632 mAh g^−1^ at 10 mA cm^−2^ (Fig. 8d) indicating efficient zinc utilization and effective electrochemical energy conversion. The long-term rechargeability of the ZAB was further investigated by continuous GCD cycling at 10 mA cm^−2^ as illustrated in Fig. 8e, the battery maintains stable charge–discharge profiles for 110 h with negligible voltage decay or any variation in the polarization gap. The decent cycling durability indicates sustained catalytic activity and durable structural stability of the air cathode during repeated ORR/OER processes. These results suggest that CoFe_2_O_4_@DP@PCNF is a promising bifunctional oxygen electrocatalyst having potential for rechargeable zinc–air battery practical applications.

**Fig. 8:**
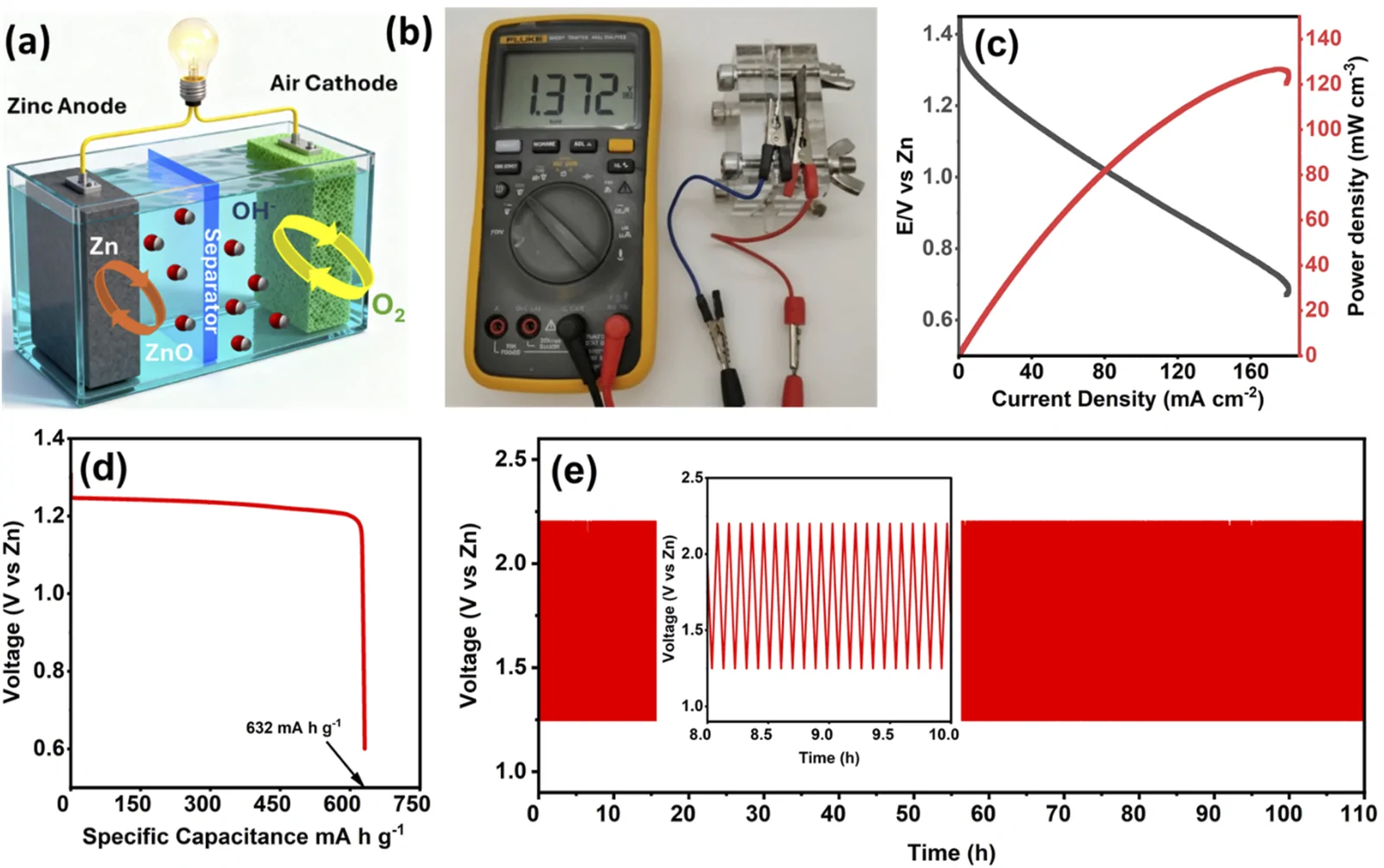
Testing of assembled ZAB utilizing CoFe_2_O_4_@DP@PCNF air-electrode. (a) Schematic illustration, (b) open-circuit potential (c) discharge polarization curve along with the corresponding power density curve, (d) specific capacity performance under a current density of 10 mA cm^−2^, (e) galvanostatic cycling stability at a current density of 10 mA cm^−2^ for of 110 hours.

## Conclusion

In summary, a catechol-assisted strategy was successfully employed to achieve the controlled growth of ultrafine CoFe_2_O_4_ nanorods uniformly anchored on porous carbon nanofibers for efficient bifunctional oxygen electrocatalysis. The introduction of dopamine-derived catechol groups enabled strong metal support coordination, resulting in a remarkable nanorod size reduction from ∼103 nm to ∼29 nm and uniform dispersion across the carbon matrix. The optimized CoFe_2_O_4_@DP@PCNF catalyst exhibited a half-wave potential of 0.77 V (*vs.* RHE) for ORR, approaching that of Pt/C (0.79 V), and achieved a current density of 10 mA cm^−2^ at 301 ± 4 mV (*vs.* RHE) for OER, surpassing RuO_2_ (410 mV). When employed as the air electrode in a rechargeable Zn–air battery, the catalyst delivered an open-circuit voltage of 1.372 V and stable up to 110 h, highlighting its excellent durability and reversibility. This study demonstrates that catechol-functionalized carbon nanofibers provide an effective platform for precise nanorod control and enhanced interfacial coupling, leading to substantial improvements in catalytic performance.

## Author contributions

Rabiya Awan: conceptualization, methodology, investigation, validation, writing – original draft. Usman Hayder: methodology, investigation. Senem Çitoğlu: methodology, investigation. Hatice Duran: resources, investigation. Salman Arshad: conceptualization, resources, supervision, funding acquisition, writing – reviewing and editing, project administration.

## Conflicts of interest

The authors declare that they have no known competing financial interests or personal relationships that could have appeared to influence the work reported in this paper.

## Supplementary Material

RA-OLF-D6RA09690E-s001

## Data Availability

The data supporting this article have been included as part of the supplementary information (SI). Supplementary information is available. See DOI: https://doi.org/10.1039/d6ra09690e.
