# Supplementary material for: Catechol-functionalized carbon nanofibers enabling ultrafine CoFe2O4 nanorods for high-performance bifunctional oxygen electrocatalysis and Zn–air batteries
Source: RSC Adv. 2026 Nov 4. Online ahead of print. doi: 10.1039/d6ra09690e (PMC13732314; doi:10.1039/d6ra09690e)
Supplement: Supplementary file 1 [file RA-OLF-D6RA09690E-s001.pdf]

Supplementary Information (SI) for RSC Advances.
This journal is © The Royal Society of Chemistry

Supplementary Information

Catechol-functionalized carbon nanofibers enabling ultrafine $CoFe_2O_4$ nanorods for high-performance bifunctional oxygen electrocatalysis and Zn-air batteries

Rabiya Javed Awan,[*a] Usman Hayder,[b] Senem Çitoğlu,[c] Hatice Duran,[c,d] and Salman Noshear Arshad[*a,e,f]

[a] Department of Chemistry and Chemical Engineering, Syed Babar Ali School of Science and Engineering, Lahore University of Management Sciences, Lahore 54792, Pakistan.

[b] Helmholtz Institute Ulm (HIU), Electrochemical Energy Storage, Helmholtzstrasse 11, 89081 Ulm, Germany

[c] Department of Materials Science & Nanotechnology Engineering, TOBB University of Economics and Technology, Söğütözü Cad. 43, Ankara, 06560, Türkiye.

[d] UNAM Institute of Materials Science and Nanotechnology, Bilkent University, 06800 Ankara, Türkiye.

[e] Department of Materials Science and Engineering, King Fahd University of Petroleum & Minerals, Dhahran 31261, Saudi Arabia.

[f] Interdisciplinary Research Center for Hydrogen Technologies and Carbon Management, King Fahd University of Petroleum & Minerals, Dhahran 31261, Saudi Arabia.

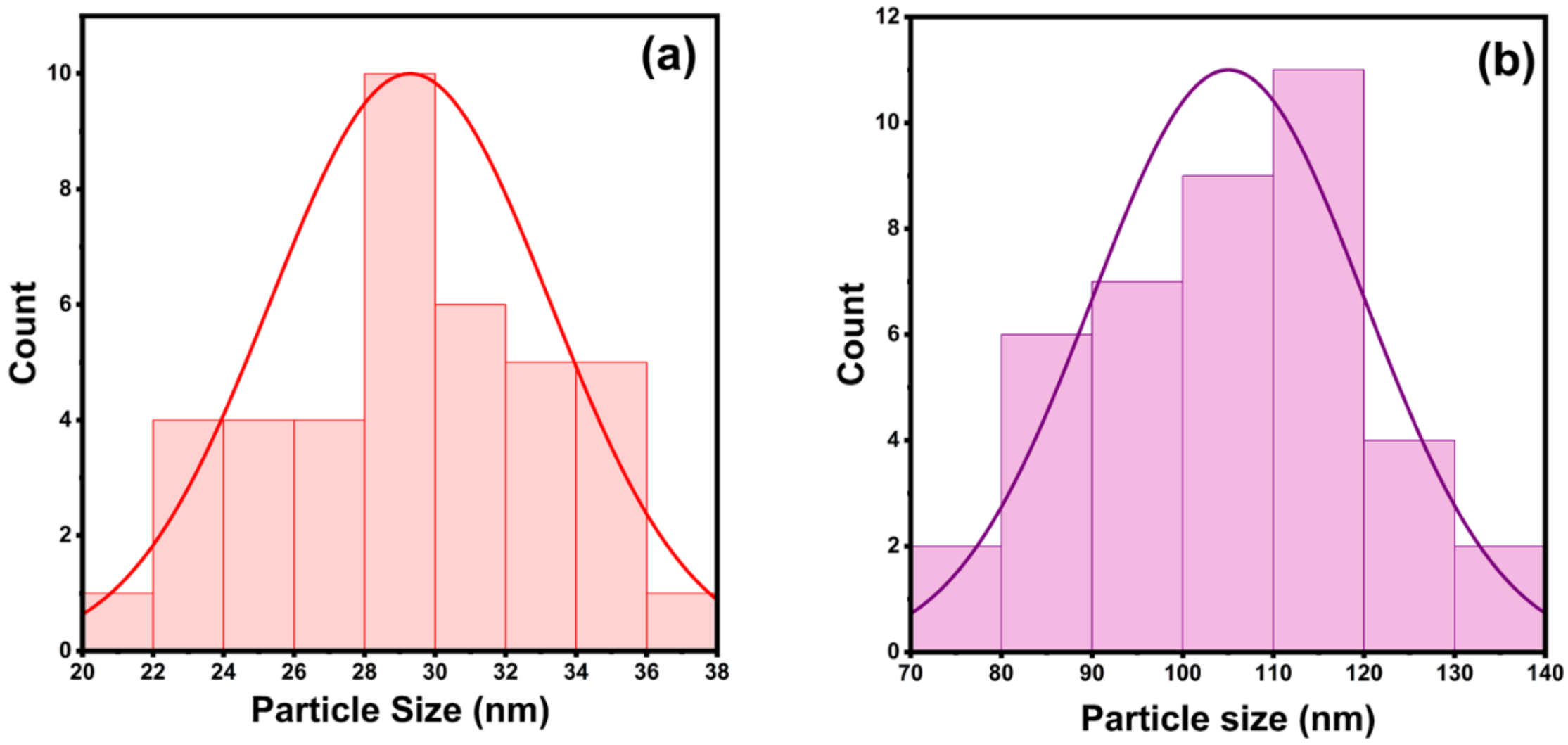

Figure S1: *Particle size distribution of $CoFe_2O_4$ grown on* ***(a)*** *catechol-functionalized PCNF, and* ***(b)*** *activated PCNF-unfunctionalized.*

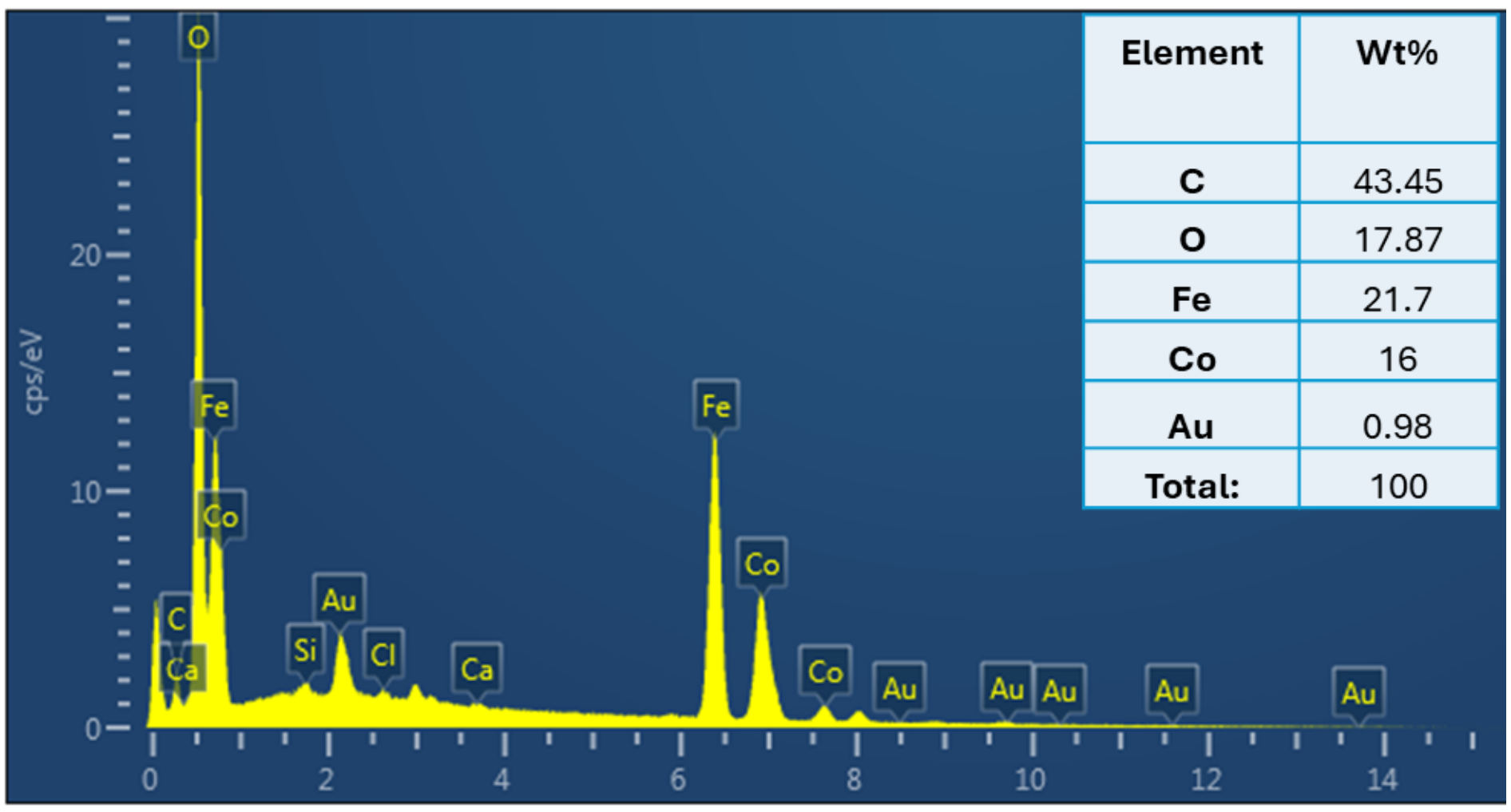

Figure S2: *EDX spectrum of $CoFe_2O_4$ grown on carboxylic acid-functionalized PCNF.*

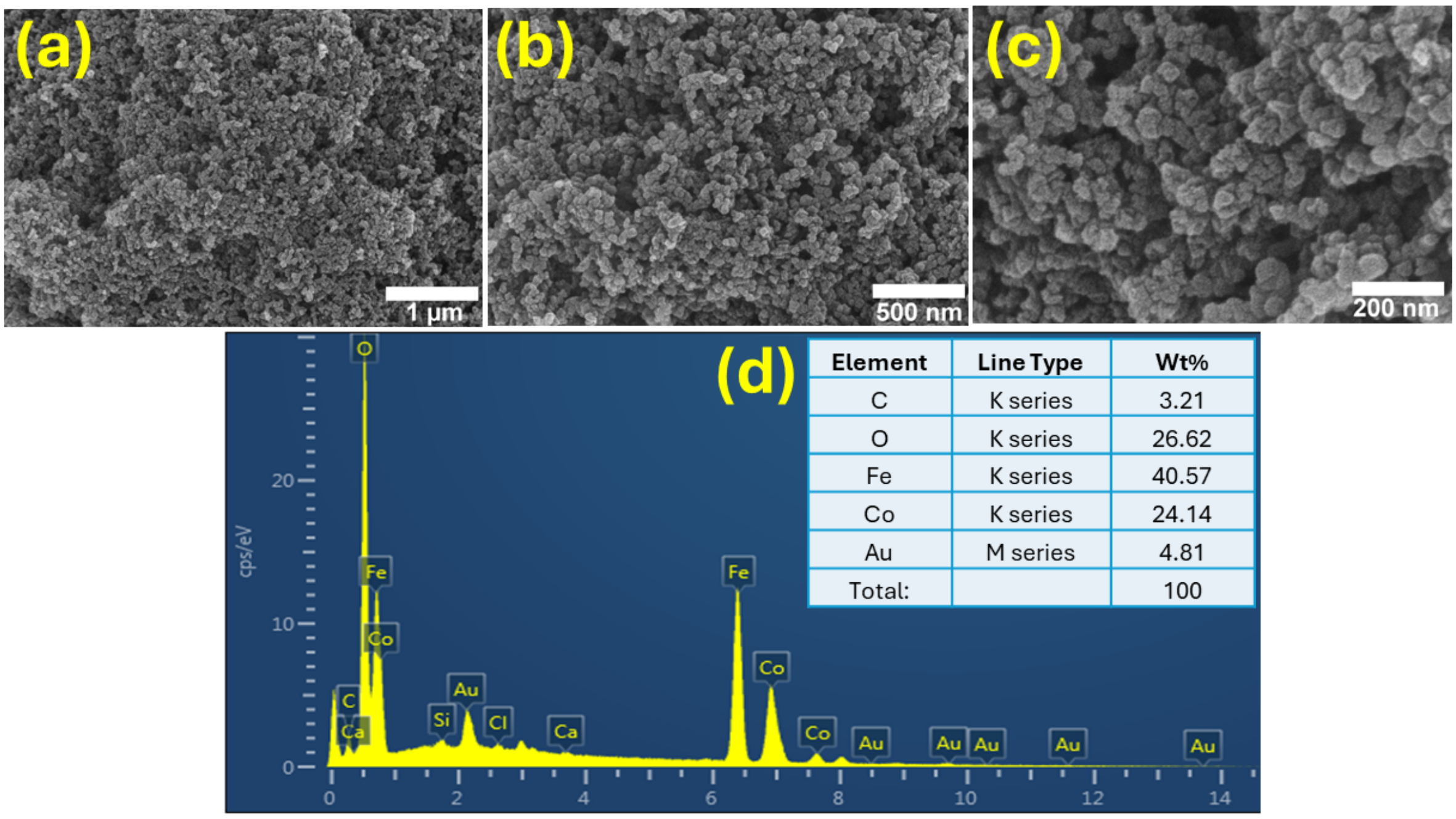

Figure S3: (a-c) SEM images of $CoFe_2O_4$ synthesized without any support, and ***(d)*** *EDX spectrum.*

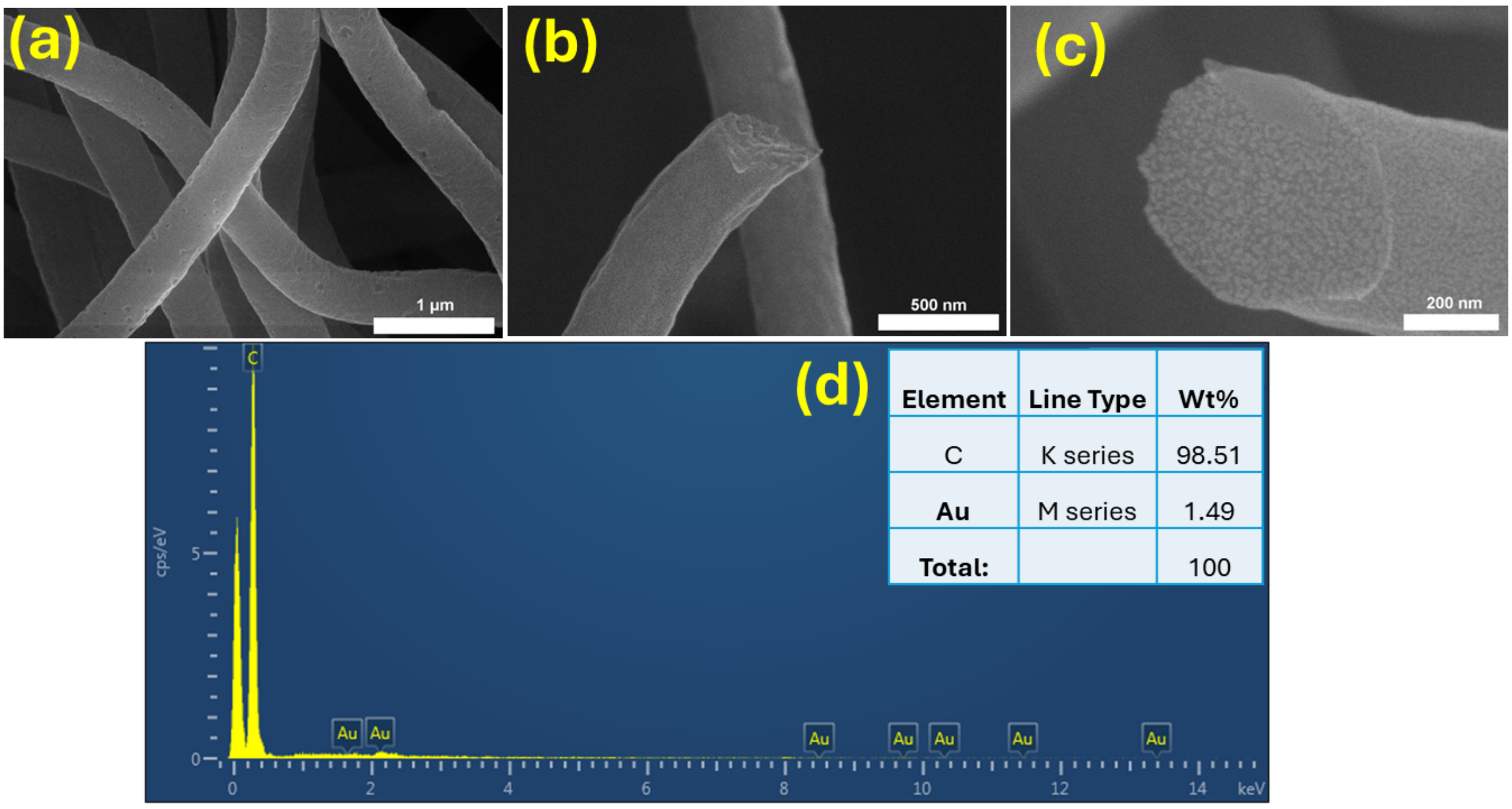

Figure S4: (a-c) SEM images of PCNF, and ***(d)*** *EDX spectrum.*

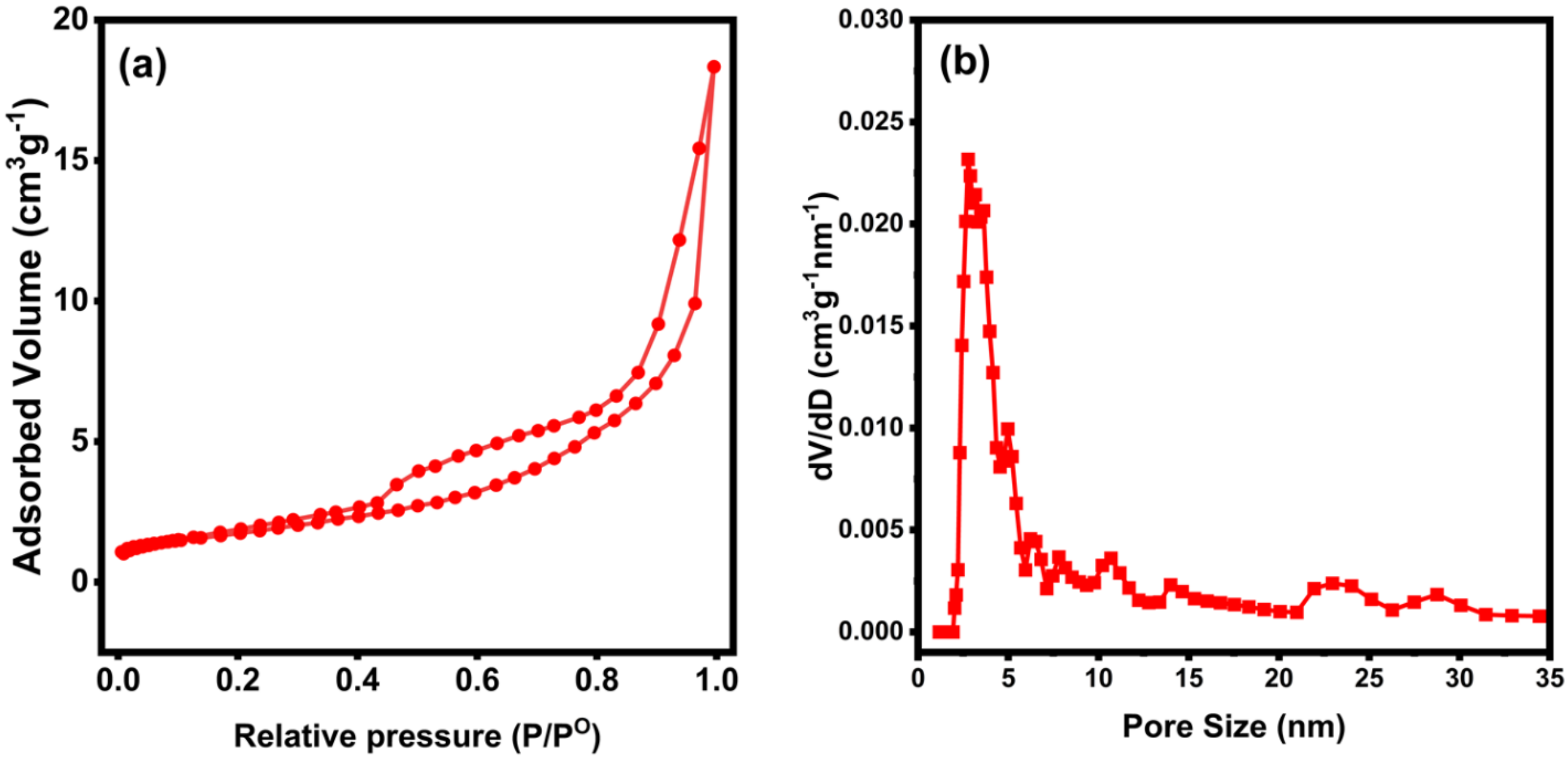

Figure S5: (a) Nitrogen adsorption-desorption isotherms of $CoFe_2O_4$@DP@PCNF, and (b) pore size distribution.

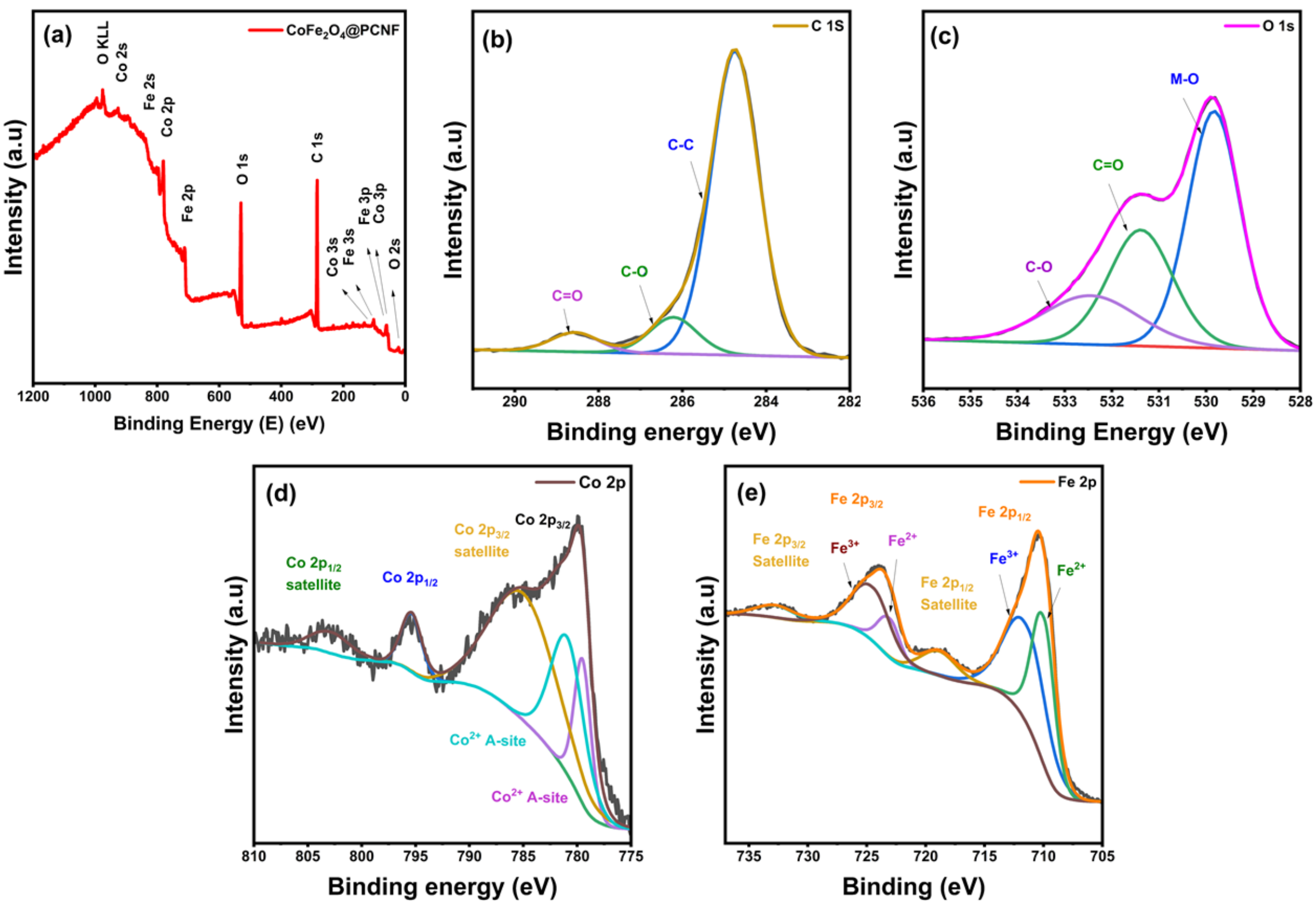

Figure S6: XPS analysis of $CoFe_2O_4$@PCNF. (a) Survey scan, and high-resolution (b) C 1s, (c) O 1s, (d) Co 2p, and (e) Fe 2p spectra.

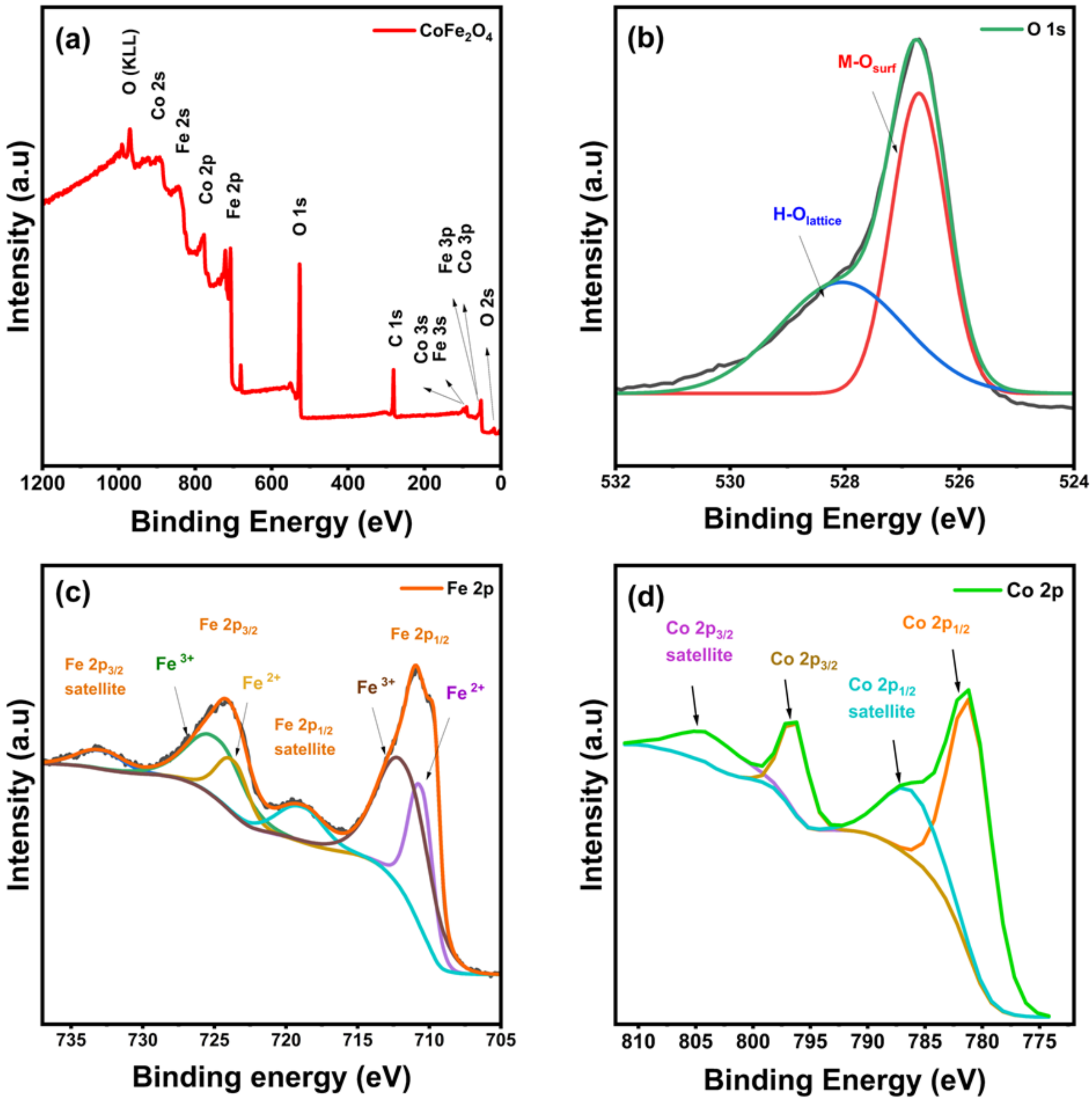

Figure S7: XPS analysis of $CoFe_2O_4$. (a) Survey scan, and high-resolution (b) O 1s, (c) Fe 2p, and (d) Co 2p spectra.

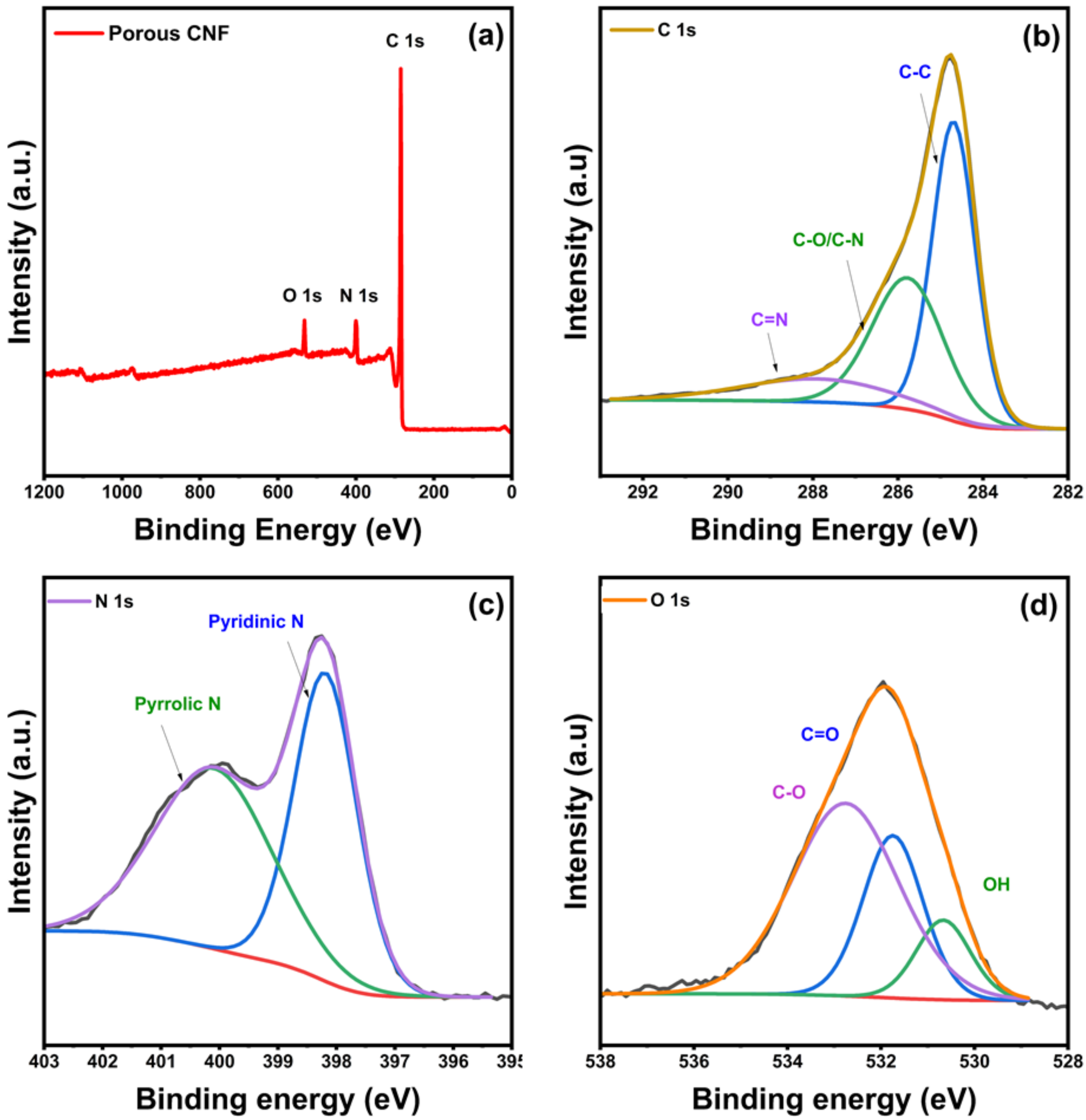

Figure S8: *XPS analysis of PCNF.* ***(a)*** *Survey scan, and high-resolution* ***(b)*** *C 1s,* ***(c)*** *N 1s,* ***(d)*** *O 1s spectra.*

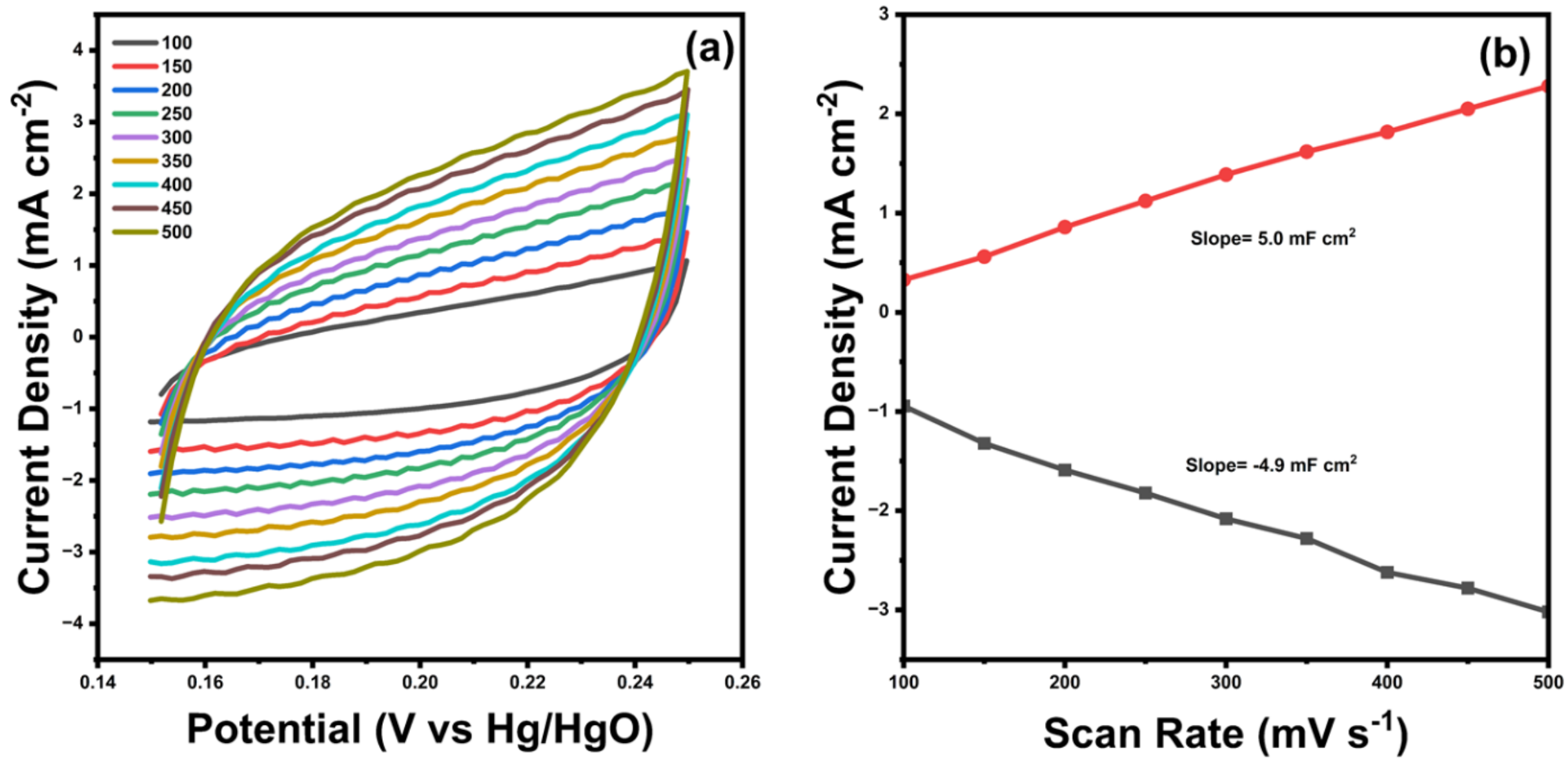

Figure S9: Cyclic voltammetry of the $CoFe_2O_4$@DP@PCNF taken in the non-faradic region at different scan rates.

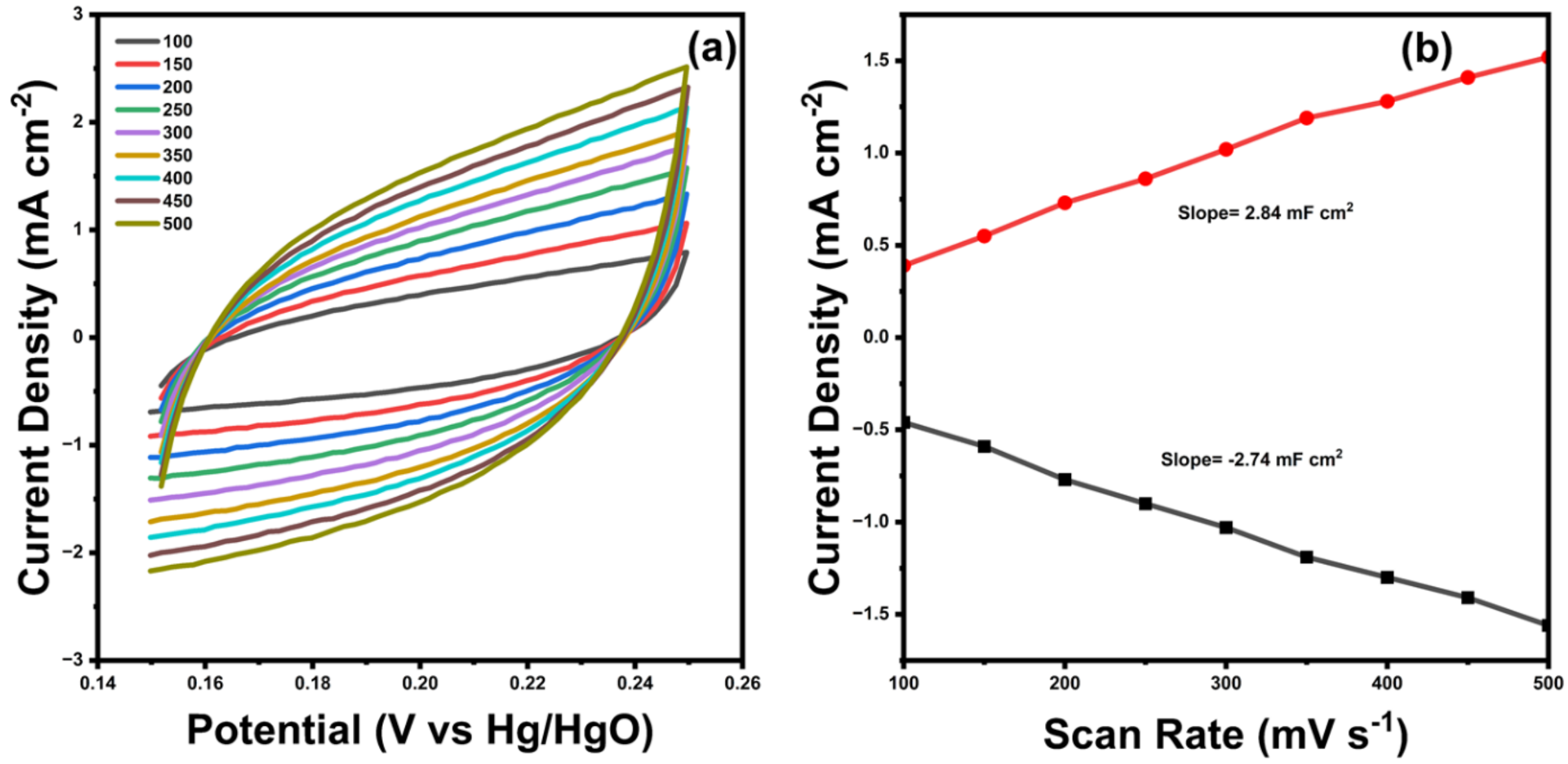

Figure S10: Cyclic voltammetry of the $CoFe_2O_4$@PCNF taken in the non-faradic region at different scan rates.

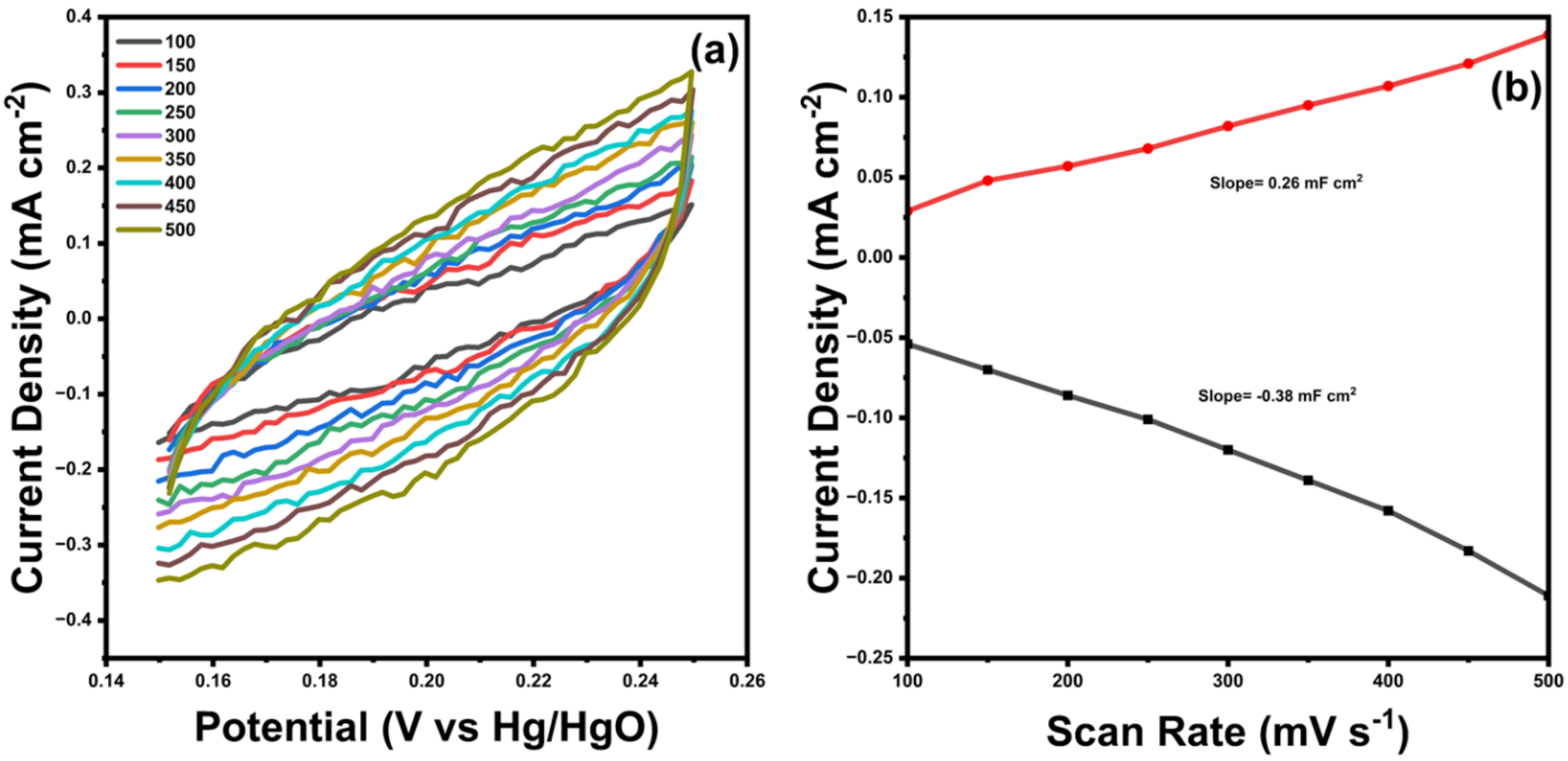

Figure S11: *Cyclic voltammetry of the $CoFe_2O_4$ taken in the non-faradic region at different scan rates.*

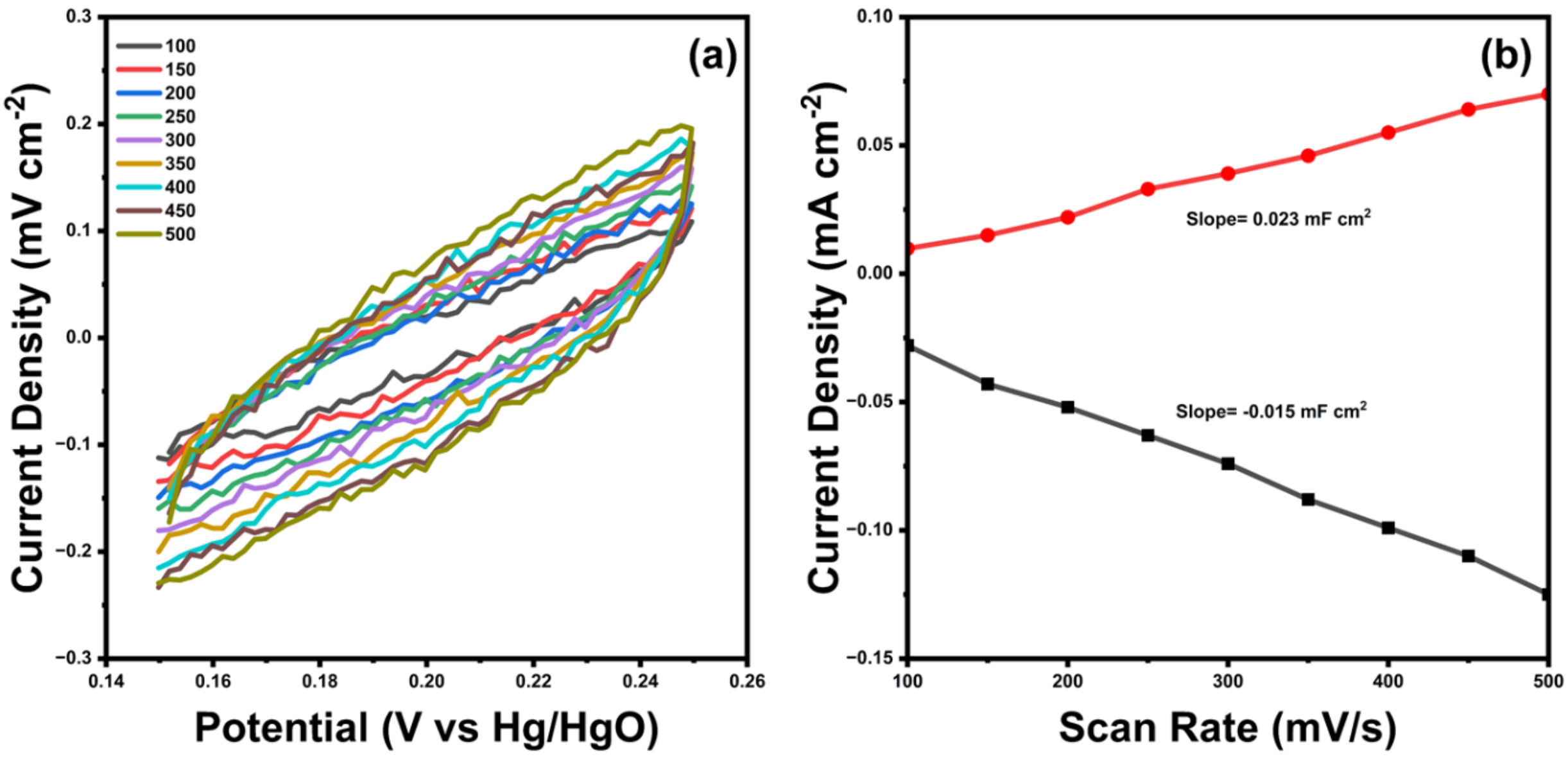

Figure S12*: Cyclic voltammetry of the PCNF taken in the non-faradic region at different scan rates.*

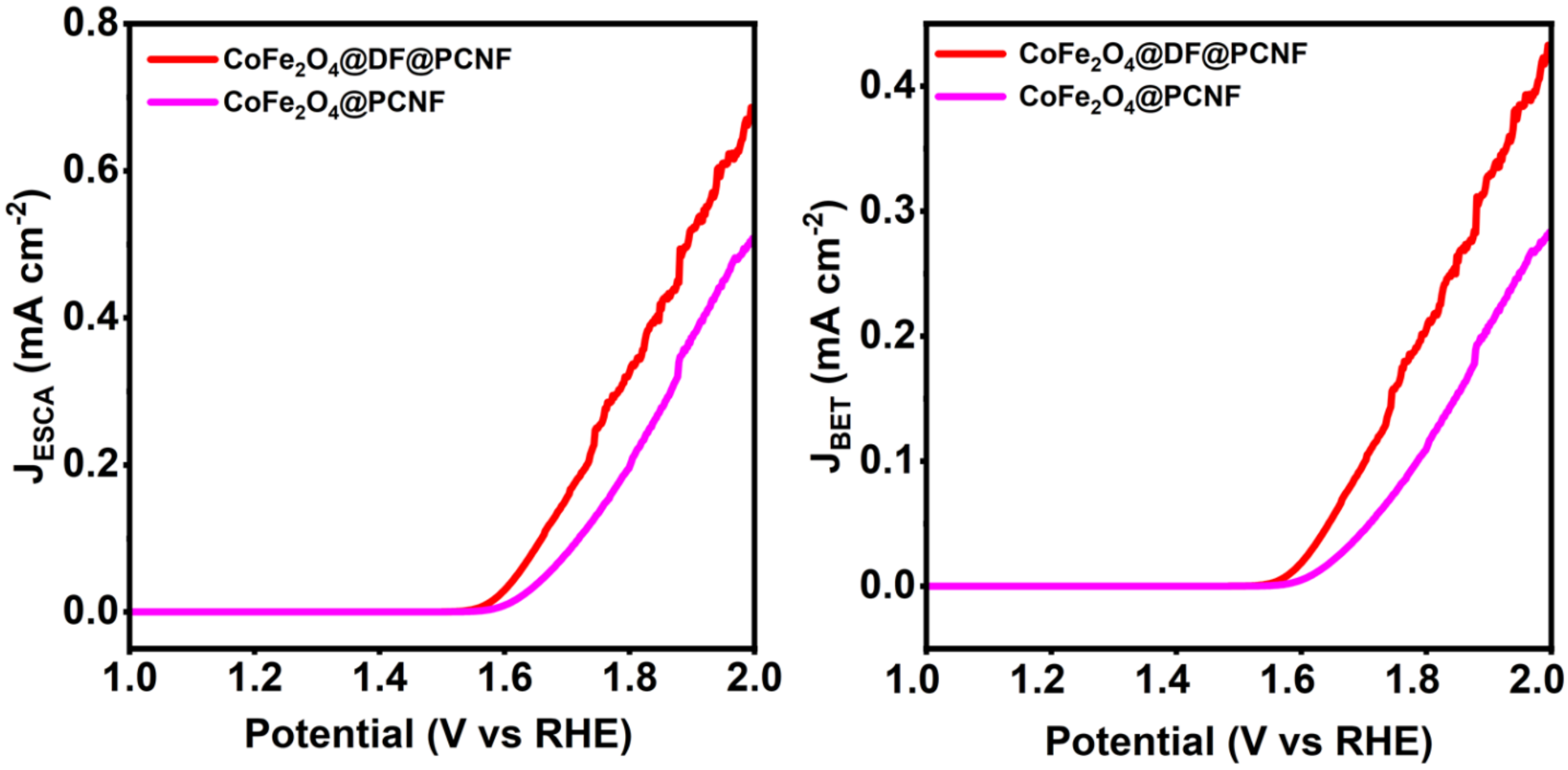

Figure S13: ***(a)*** *ESCA normalized LSV, and* ***(b)*** *BET normalized LSV for OER.*

Table S1: *Comparison of OER performance of the $CoFe_2O_4$@DP@PCNF catalyst with the literature for Co-based materials tested on glassy carbon in 1M KOH.*

No.	Catalyst	Electrolyte	OER over potential (mV) at 10 mA cm^{-2}	Tafel Slope (mV dec^{-1})	Ref.
1	Co_3O_4/LIG	1M KOH	340	40	[1]
2	$NiCo_{2.148}O_4$ PN	1M KOH	290	50	[2]
3	$NiCo_2O_4$ nanocrystal@MXene	1M KOH	310	64	[3]
4	Co_3O_4–$NiCo_2O_4$/N-RGO	1M KOH	390	124	[4]
5	Co_3O_4@WO_3	1M KOH	339	93	[5]
6	Co_3O_4/N-rGO	1M KOH	490	110	[6]
7	$Co_2P/Mo_2C/Mo_3Co_3C$@C	1M KOH	362	82	[7]
8	Fe_3O_4@Co_9S_8/rGO	1M KOH	320	54	[8]
9	Co_3O_4/reduced GO	1M KOH	310	47	[9]
10	$Co_3O_4/CoMoO_4$-50	1M KOH	318	63	[10]
11	NiCoP/C nanoboxes	1M KOH	330	96	[11]
12	$Fe_{0.5}Ni_{0.5}Co_2O_4$	1M KOH	350	27	[12]
13	Co_9S_8/NSCNFs-850	1M KOH	302	54	[13]
14	$Ni_xCo_{3-x}O_4$ nanowires	1M KOH	337	75	[14]
15	Co-NC/CNT	1M KOH	354	78	[15]
16	NiCoS/$Ti_3C_2T_X$	1M KOH	365	58	[16]
17	CoMo–H	1M KOH	312	69	[17]
18	Zn-doped $CoSe_2$/CFC	1M KOH	356	88	[18]
19	CoP_3 CPs/CFP	1M KOH	343	76	[19]
20	Commercial RuO_2	1M KOH	400	113	[20]

21	$CoFe_2O_4$@DP@PCNF	1M KOH	301	71	This work

Normalization of the polarization curves

The polarization curves were normalized to the ECSA according to the following equation:

$$j_{ECSA}\ (mA/cm^2) = \frac{\square \times 1000}{ECSA} \qquad S1$$

where i (A) is the current, and ECSA is the electrochemical active surface area (cm^2) of the material.

The polarization curves were normalized to the BET surface area according to the following equation:

$$j_{BET}\ (mA/cm^2) = \frac{\square \times 1000}{S \times BET\ surface\ area \times m_{loading}} \qquad S2$$

where i (A) is the current, S is the geometric area of glassy carbon, BET_{area} is calculated from the nitrogen absorption-desorption isotherms, and $m_{loading}$ is the loading of catalyst on glassy carbon (0.001 g cm^{-2}).

Faradaic Efficiency Calculation:

The faradaic efficiency of the catalyst was calculated by comparing the amount of moles of oxygen produced experimentally with the theoretically calculated value. Equation S3 was used to compute theoretical value at a constant current value of 10mA cm^{-2}.

$$n = \frac{Q}{4f} \qquad S3$$

where Q is the charge transfer, F is the Faraday constant, and n is the number of moles of oxygen gas produced.

$$\text{Faradaic efficiency (F.E)} = \frac{n\ (experimental)}{n\ (theoratical)} \times 100 \qquad S4$$

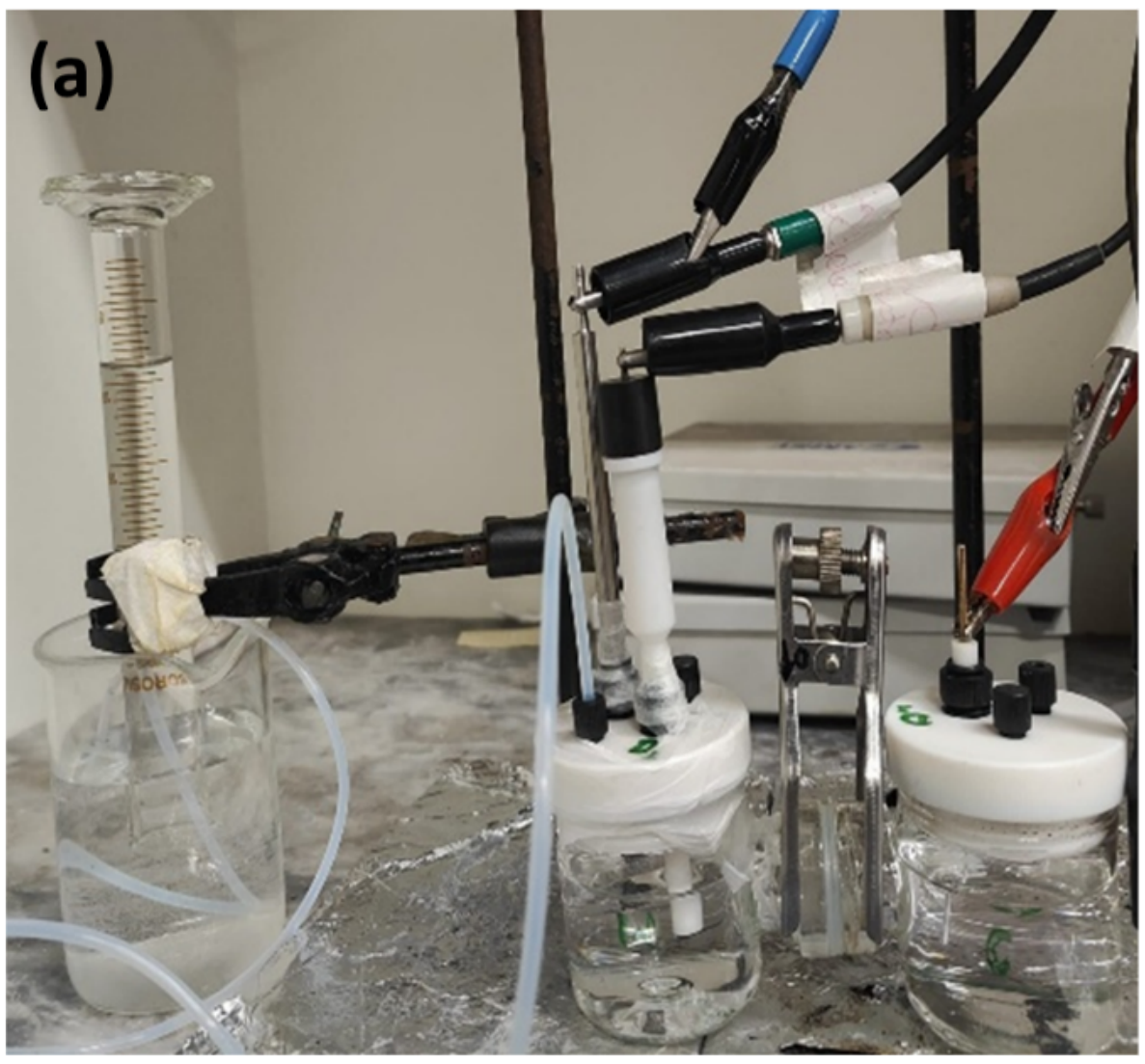

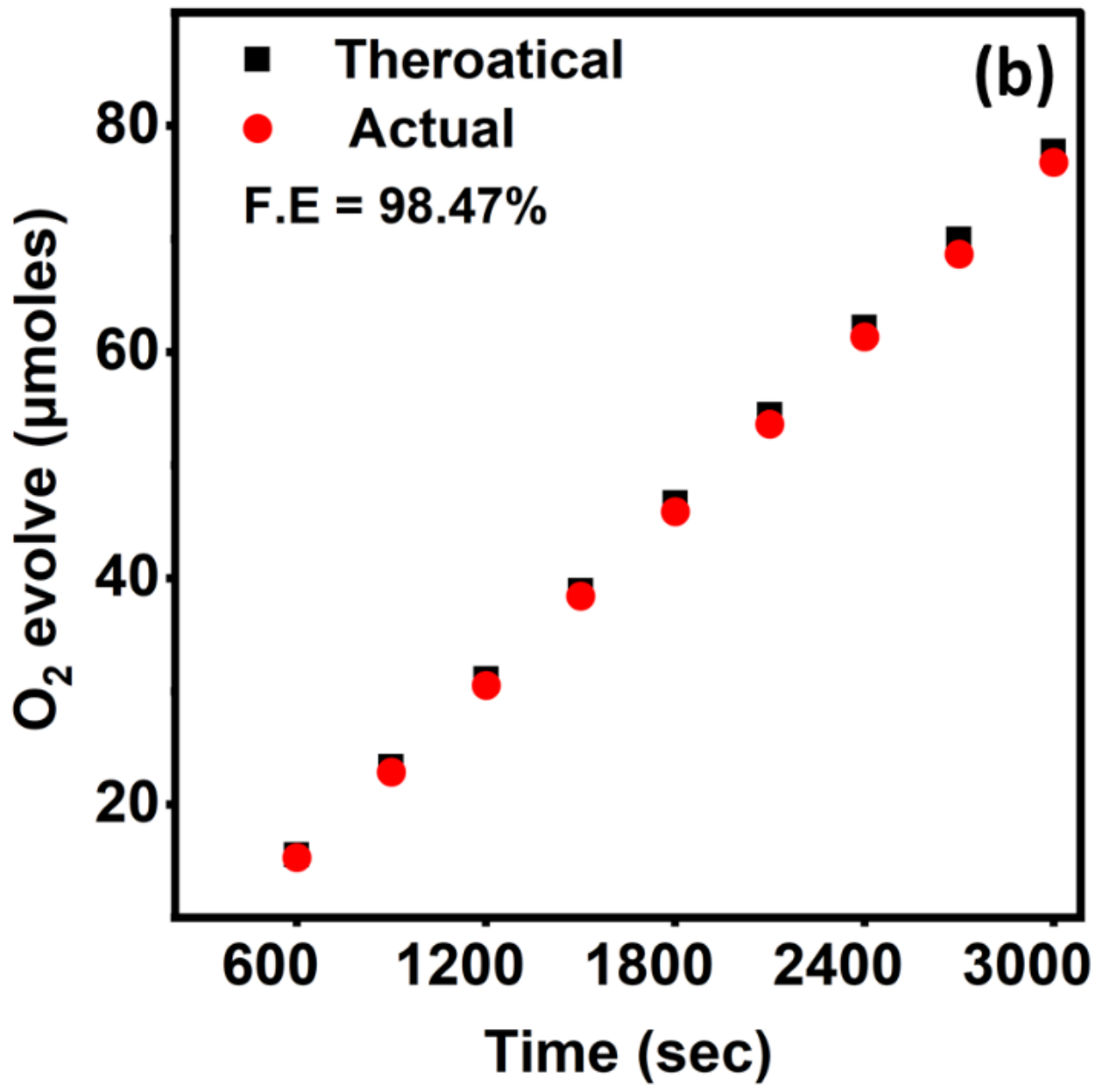

Figure S14: (a) H-cell setup for water displacement method, and (b) Faradic efficiency of O_2 at constant current density of 10 mA cm^{-2}.

Turnover Frequency Calculations:

$$TOF = i \times \frac{N_A}{A} \times F \times n \times \square \qquad S5$$

Where, i = current in Ampere, N_A= Avogadro number, A = Geometrical surface area of the electrode (1 cm^2), F = Faraday constant, n = Number of electrons, □□ = Surface concentration of atoms obtained by the integration of the redox peak of OER polarization curve.

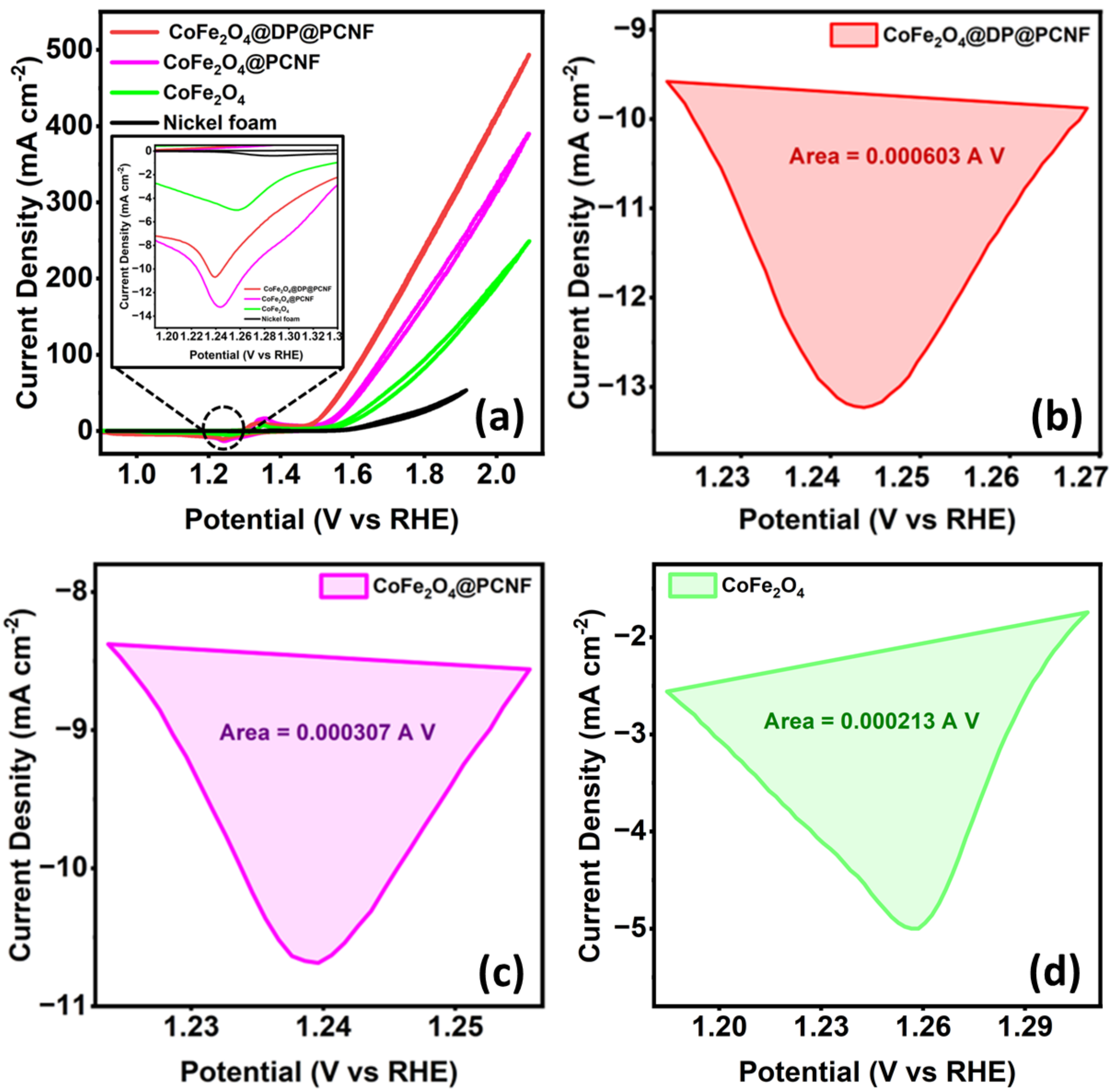

Figure S15: (a) *CV curves taken at scan rate of 5mV s^{-1} of all the catalysts to calculate TOF, and* ***(b-d)*** *absolute area of CoFe$_2$O$_4$@DP@PCNF, CoFe$_2$O$_4$@PCNF, and CoFe$_2$O$_4$, respectively.*

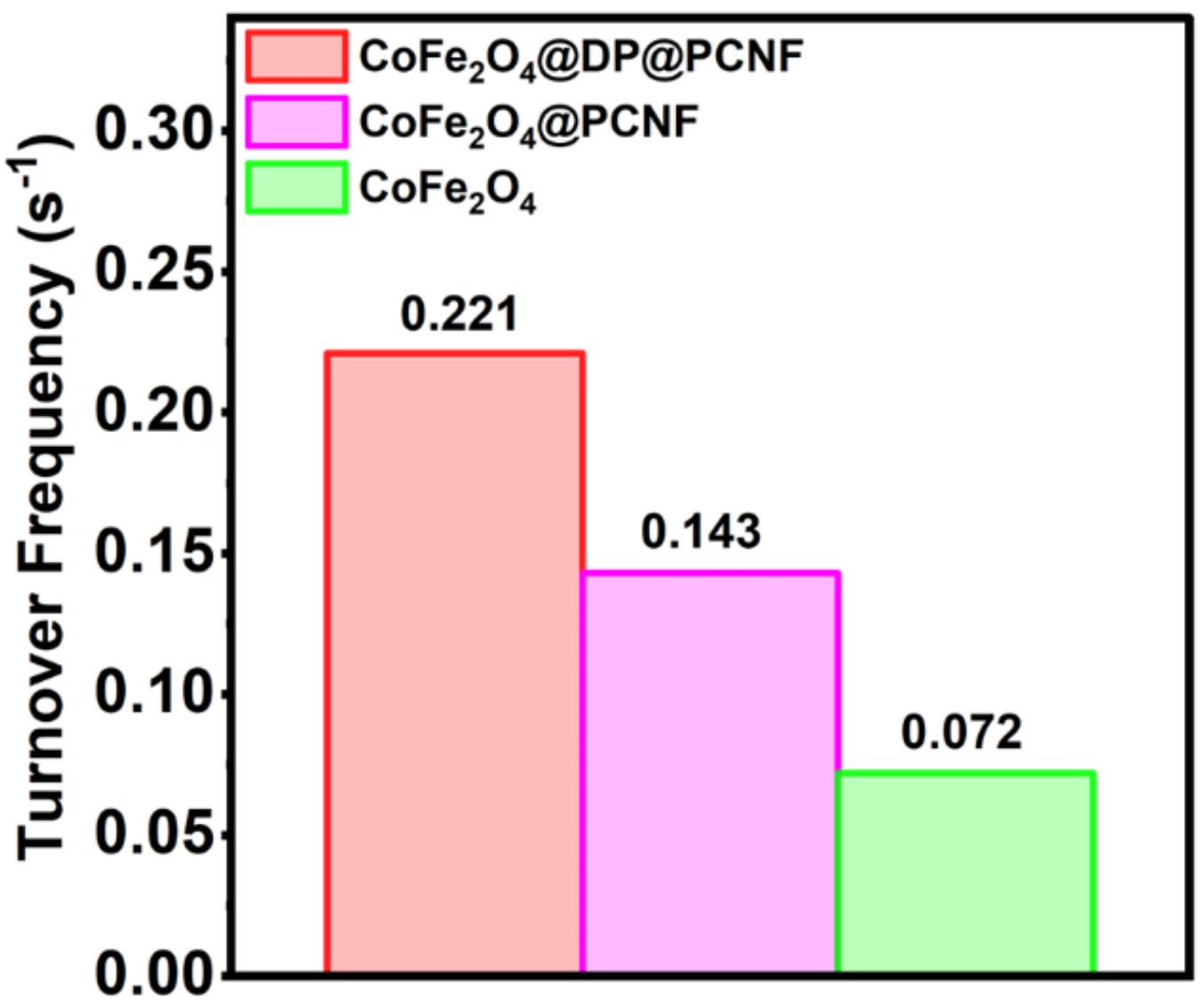

Figure S16: TOF values of the catalysts.

Activation Energy Calculations:

For electrocatalytic activation energy calculations, the Arrhenius form is used [21]:

$$j = Ae^{-E_a/RT} \quad \text{S6}$$

Taking the natural logarithm:

$$\ln j = \ln A - \frac{E_a}{R}\left(\frac{1}{T}\right) \quad \text{S7}$$

So, the slope from j vs $\frac{1}{T}$ plot is:

slope = $-\frac{E_a}{R}$ Since, R = 8.314 J mol^{-1} K^{-1}

$$E_a (\text{kJ mol}^{-1}) = \frac{-(\text{slope}) \times 8.314}{1000}$$

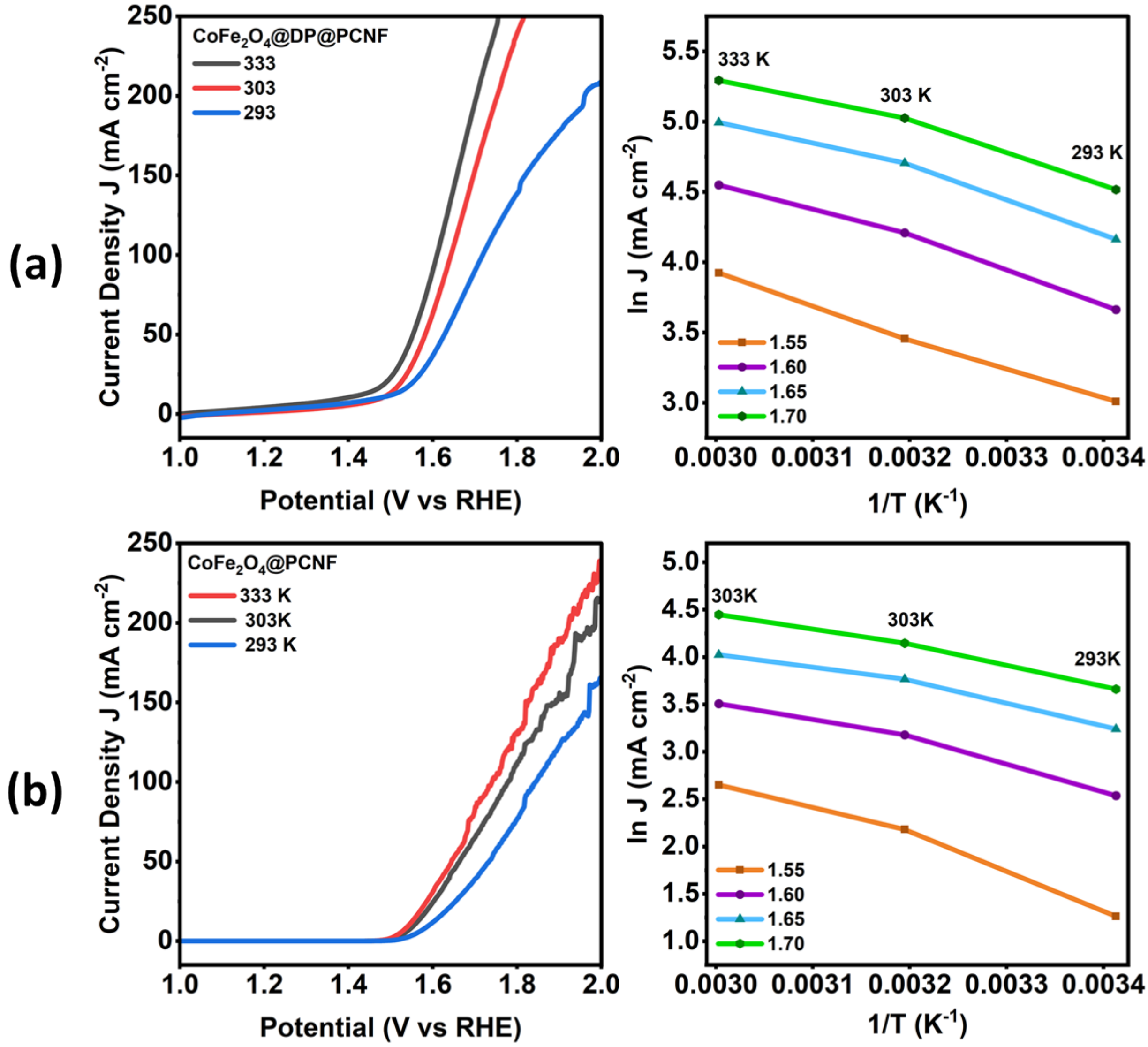

Figure S17: *Temperature-dependent OER polarization curves of **(a)** $CoFe_2O_4$@DP@PCNF and **(b)** $CoFe_2O_4$@PCNF, along with their corresponding Arrhenius plots of ln(j) versus 1/T.*

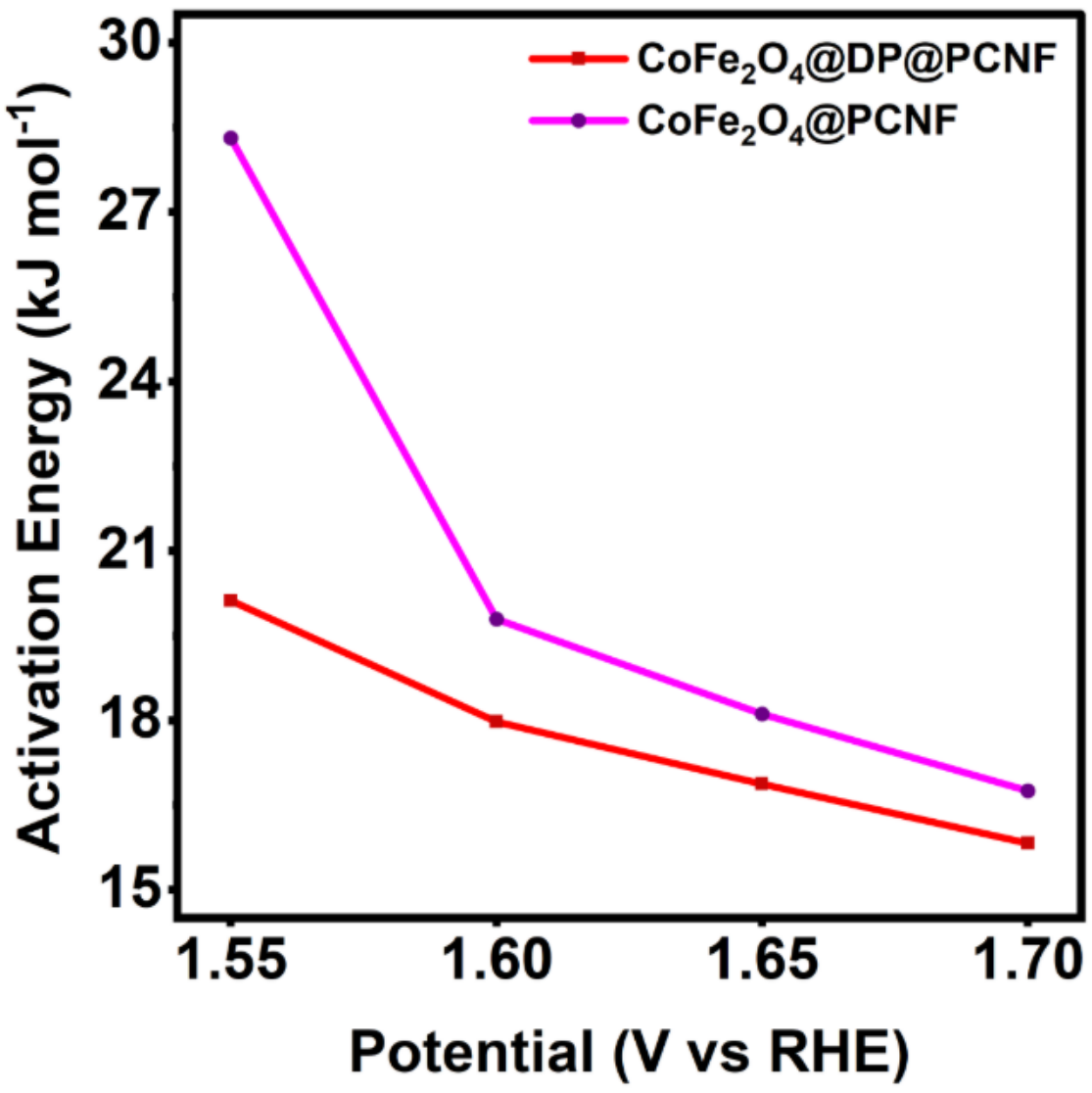

Figure S18:*Activation energies (E_a) for OER using $CoFe_2O_4$@PCNF and $CoFe_2O_4$@DP@PCNF catalysts.*

Table S2: *The onset potential (E_{onset}), half-wave potential ($E_{1/2}$), and current densities ($J_{0.4}V$) acquired from the LSV measurements for all the catalysts.*

	Onset potential (E_{onset})	Half-wave potential ($E_{1/2}$)	Current densities ($J_{0.4}V$)
$CoFe_2O_4$@DP@PCNF	0.90	0.77	0.85
$CoFe_2O_4$@PCNF	0.82	0.70	0.77
$CoFe_2O_4$	0.79	0.67	0.75
PCNF	0.74	0.62	0.66

Table S3: *Comparison of ORR performance of the $CoFe_2O_4$@DP@PCNF catalyst with the literature for Co-based materials.*

No.	Catalyst	Electrolyte (Concentration of KOH)	ORR Half-wave potential $E_{1/2}$ (V)	References
1	FB-CoFe-800	0.1M	0.76	[22]
2	FNCM-CNT@CNF	0.1M	0.80	[23]
3	$CoFe_2O_4$ nanoparticles	0.1M	0.67	[24]
4	Co_3O_4/CNTs	0.1M	0.83	[25]
5	NiCoSn alloy	1M	0.72	[26]
6	Co_3O_4/LIG	0.1M	0.72	[1]
7	CoFe/N-GCT	0.1M	0.79	[27]
8	$NiCo_2O_4$/Co,N-CNT	0.1M	0.86	[28]
9	$NiCo_2O_4$ NCs	0.1M	0.67	[28]
10	Co_3O_4/Ag@NrGO	0.1M	0.73	[29]
11	Co@Co_3O_4@NC-900	0.1M	0.75	[30]
12	$NiCo_{2.148}O_4$ PN	0.1M	0.65	[2]
13	$NiCo_2O_4$ @MXene	0.1M	0.70	[3]
14	Co_3O_4–$NiCo_2O_4$/N-RGO	0.1M	0.79	[4]
15	Co_3O_4@WO_3	1M	0.76	[5]
16	FeNi-CNT@CNF	0.1M	0.80	[31]
17	Fe–Co–Ni MOF	1M	0.75	[32]
18	Co-MOF	1M	0.70	[33]
19	Co_3O_4/N-rGO	1M	0.79	[6]
20	Commercial Pt/C	0.1M	0.79	[20]
**21**	**$CoFe_2O_4$@DP@PCNF**	**1M**	**0.77**	**This Work**

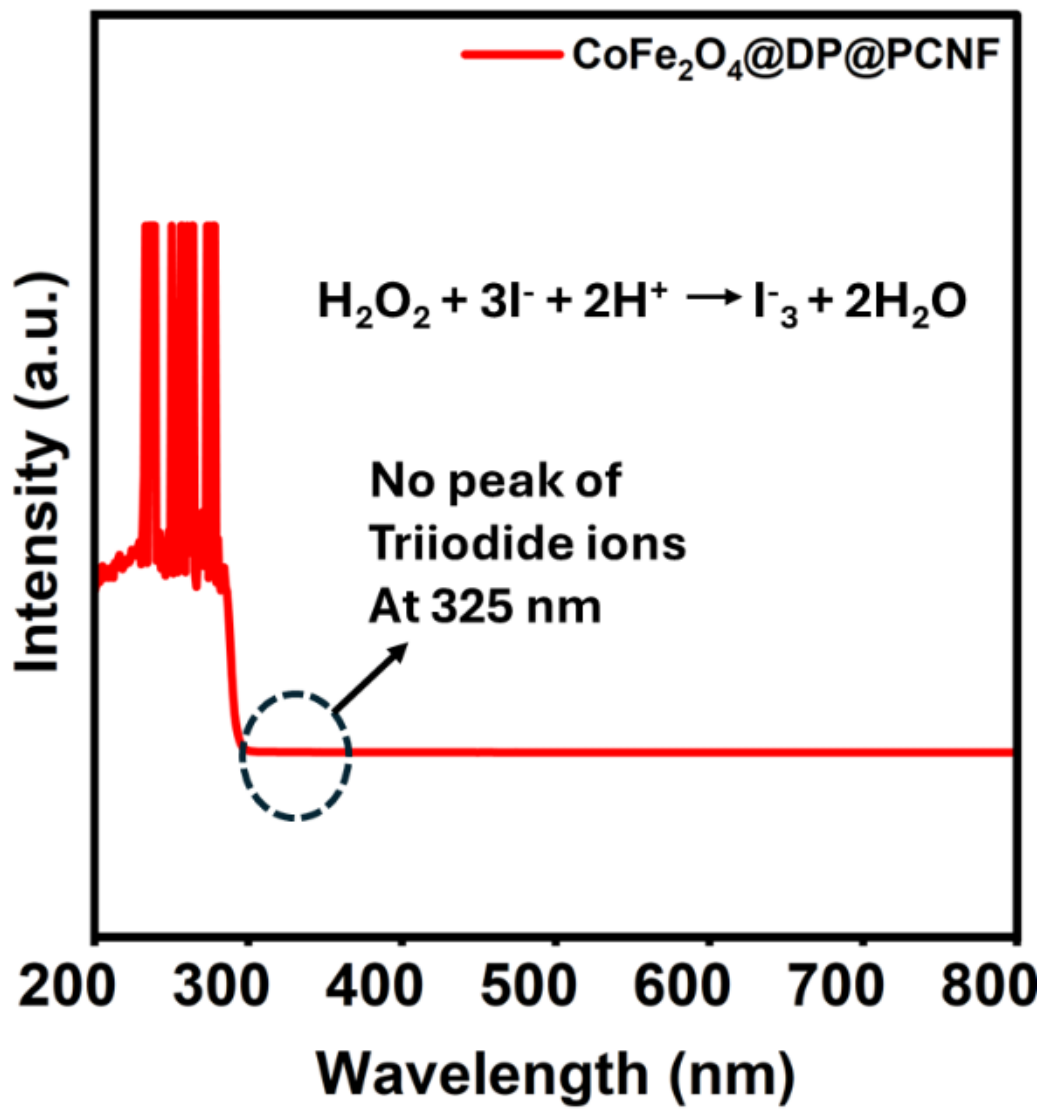

Figure S19: *UV-vis analysis for the detection of H_2O_2.*

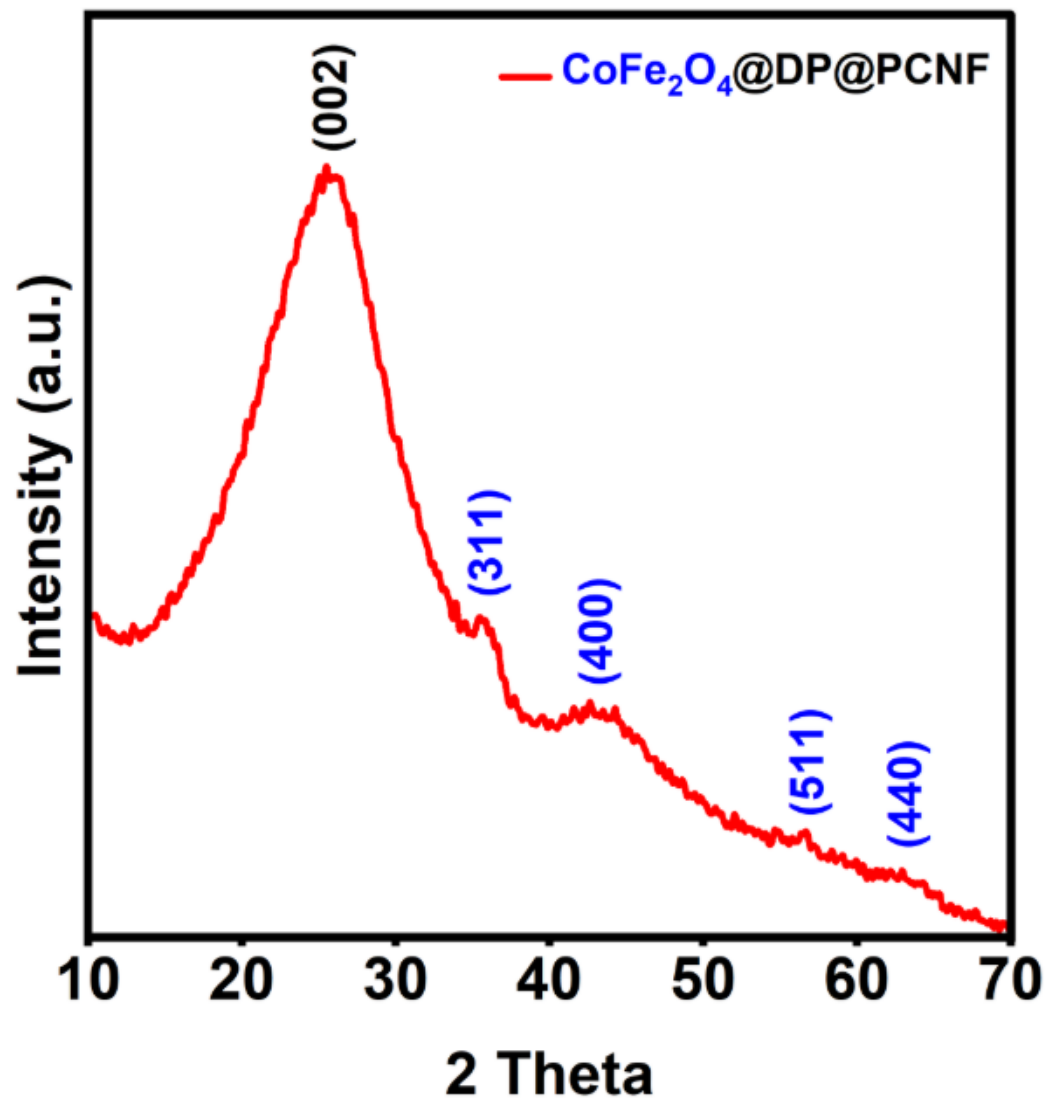

Figure S20: *Post-stability XRD of $CoFe_2O_4$@DP@PCNF catalyst.*

References:

1. Ren, M., J. Zhang, and J.M. Tour, *Laser-induced graphene synthesis of Co3O4 in graphene for oxygen electrocatalysis and metal-air batteries.* Carbon, 2018. **139**: p. 880-887.
2. Yin, J., et al., *NiCo2O4-based nanosheets with uniform 4 nm mesopores for excellent Zn–air battery performance.* Advanced Materials, 2020. **32**(39): p. 2001651.
3. Lei, H., et al., *Strongly coupled NiCo2O4 nanocrystal/MXene hybrid through in situ Ni/Co–F bonds for efficient wearable Zn–air batteries.* ACS Applied Materials & Interfaces, 2020. **12**(40): p. 44639-44647.

4. Zhu, Z., et al., *Co3O4–NiCo2O4 hybrid nanoparticles anchored on N-Doped reduced graphene oxide nanosheets as an efficient catalyst for Zn–air batteries.* Energy & Fuels, 2021. **35**(5): p. 4550-4558.
5. Li, A., Y. Wang, and C. Cheng, *Three-dimensionally ordered Co3O4@ WO3 composite arrays as a binder-free air cathode for high-performance Zn-air batteries.* Materials Today Nano, 2023. **22**: p. 100339.
6. Li, Y., et al., *Atomically thin mesoporous Co3O4 layers strongly coupled with N-rGO nanosheets as high-performance bifunctional catalysts for 1D knittable zinc–air batteries.* Advanced Materials, 2018. **30**(4): p. 1703657.
7. Li, X., et al., *Ternary hybrids as efficient bifunctional electrocatalysts derived from bimetallic metal–organic-frameworks for overall water splitting.* Journal of Materials Chemistry A, 2018. **6**(14): p. 5789-5796.
8. Yang, J., et al., *Fe3O4-decorated Co9S8 nanoparticles in situ grown on reduced graphene oxide: a new and efficient electrocatalyst for oxygen evolution reaction.* Advanced Functional Materials, 2016. **26**(26): p. 4712-4721.
9. Liang, Y., et al., *Co3O4 nanocrystals on graphene as a synergistic catalyst for oxygen reduction reaction.* Nature materials, 2011. **10**(10): p. 780-786.
10. Zhang, L., et al., *Hollow POM@ MOF hybrid-derived porous Co 3 O 4/CoMoO 4 nanocages for enhanced electrocatalytic water oxidation.* Journal of Materials Chemistry A, 2018. **6**(4): p. 1639-1647.
11. He, P., X.Y. Yu, and X.W. Lou, *Carbon-incorporated nickel–cobalt mixed metal phosphide nanoboxes with enhanced electrocatalytic activity for oxygen evolution.* Angewandte Chemie, 2017. **129**(14): p. 3955-3958.
12. Yan, K.-L., et al., *Ternary mixed metal Fe-doped NiCo2O4 nanowires as efficient electrocatalysts for oxygen evolution reaction.* Applied Surface Science, 2017. **416**: p. 371-378.
13. Huang, Z.-Q., et al., *A mesoporous C, N-co doped Co-based phosphate ultrathin nanosheet derived from a phosphonate-based-MOF as an efficient electrocatalyst for water oxidation.* Catalysis Science & Technology, 2019. **9**(17): p. 4718-4724.
14. Yan, X., et al., *From Water Oxidation to Reduction: Transformation from Ni x Co3–x O4 Nanowires to NiCo/NiCoO x Heterostructures.* ACS applied materials & interfaces, 2016. **8**(5): p. 3208-3214.
15. Yang, F., et al., *A cobalt-based hybrid electrocatalyst derived from a carbon nanotube inserted metal–organic framework for efficient water-splitting.* Journal of Materials Chemistry A, 2016. **4**(41): p. 16057-16063.
16. Zou, H., et al., *Metal–organic framework-derived nickel–cobalt sulfide on ultrathin mxene nanosheets for electrocatalytic oxygen evolution.* ACS applied materials & interfaces, 2018. **10**(26): p. 22311-22319.
17. Lyu, F., et al., *Self-templated fabrication of CoO–MoO2 nanocages for enhanced oxygen evolution.* Advanced Functional Materials, 2017. **27**(34): p. 1702324.

18. Dong, Q., et al., *MOF-derived Zn-doped CoSe2 as an efficient and stable free-standing catalyst for oxygen evolution reaction.* ACS applied materials & interfaces, 2016. **8**(40): p. 26902-26907.
19. Wu, T., et al., *Three-dimensional metal–organic framework derived porous CoP 3 concave polyhedrons as superior bifunctional electrocatalysts for the evolution of hydrogen and oxygen.* Physical Chemistry Chemical Physics, 2017. **19**(3): p. 2104-2110.
20. Aziz, I., et al., *Growth of carbon nanotubes over carbon nanofibers catalyzed by bimetallic alloy nanoparticles as a bifunctional electrode for Zn–air batteries.* RSC Advances, 2023. **13**(17): p. 11591-11599.
21. Duan, Y., et al., *Revealing the impact of electrolyte composition for Co-based water oxidation catalysts by the study of reaction kinetics parameters.* ACS Catalysis, 2020. **10**(7): p. 4160-4170.
22. Meng, H., et al., *CoFe/N, S–C Featured with Graphitic Nanoribbons and Multiple CoFe Nanoparticles as Highly Stable and Efficient Electrocatalysts for the Oxygen Reduction Reaction.* ACS omega, 2021. **6**(16): p. 11059-11067.
23. Aziz, I., et al., *Hierarchically Confined FeNiCoMn Alloy Nanoparticles Enable Durable Bifunctional Air Cathodes for Zinc-Air Batteries.* Chemical Communications, 2026.
24. Yin, J., et al., *CoFe2O4 nanoparticles as efficient bifunctional catalysts applied in Zn–air battery.* Journal of Materials Research, 2018. **33**(5): p. 590-600.
25. Ahmed, M.S., B. Choi, and Y.-B. Kim, *Development of highly active bifunctional electrocatalyst using Co3O4 on carbon nanotubes for oxygen reduction and oxygen evolution.* Scientific Reports, 2018. **8**(1): p. 2543.
26. Sajeev, A., et al., *Trimetallic non-noble NiCoSn alloy as an efficient electrocatalyst towards methanol oxidation and oxygen reduction reactions.* Journal of Colloid and Interface Science, 2023. **637**: p. 363-371.
27. Liu, X., et al., *A stable bifunctional catalyst for rechargeable zinc–air batteries: iron–cobalt nanoparticles embedded in a nitrogen-doped 3D carbon matrix.* Angewandte Chemie International Edition, 2018. **57**(49): p. 16166-16170.
28. Li, J., et al., *ZIF-67 as continuous self-sacrifice template derived NiCo2O4/Co, N-CNTs nanocages as efficient bifunctional electrocatalysts for rechargeable Zn–air batteries.* ACS Sustainable Chemistry & Engineering, 2018. **6**(8): p. 10021-10029.
29. Wang, Q., et al., *One-Pot Synthesis of Co3O4/Ag Nanoparticles Supported on N-Doped Graphene as Efficient Bifunctional Oxygen Catalysts for Flexible Rechargeable Zinc–Air Batteries.* Chemistry–A European Journal, 2018. **24**(55): p. 14816-14823.
30. Guo, Z., et al., *In situ encapsulation of core–shell-structured Co@ Co 3 O 4 into nitrogen-doped carbon polyhedra as a bifunctional catalyst for rechargeable Zn–air batteries.* Journal of Materials Chemistry A, 2018. **6**(4): p. 1443-1453.
31. Aziz, I., et al., *Growth of carbon nanotubes over carbon nanofibers catalyzed by bimetallic alloy nanoparticles as a bifunctional electrode for Zn–air batteries.* RSC advances, 2023. **13**(17): p. 11591-11599.

32. Shahbazi Farahani, F., et al., *Trilayer metal–organic frameworks as multifunctional electrocatalysts for energy conversion and storage applications.* Journal of the American Chemical Society, 2022. **144**(8): p. 3411-3428.

33. Chen, G., et al., *Cobalt-based metal–organic framework nanoarrays as bifunctional oxygen electrocatalysts for rechargeable Zn-Air batteries.* Chemistry–A European Journal, 2018. **24**(69): p. 18413-18418.
